# The tropical rain belts with an annual cycle and a continent model intercomparison project: TRACMIP

**DOI:** 10.1002/2016MS000748

**Published:** 2016-11-16

**Authors:** Aiko Voigt, Michela Biasutti, Jacob Scheff, Jürgen Bader, Simona Bordoni, Francis Codron, Ross D. Dixon, Jeffrey Jonas, Sarah M. Kang, Nicholas P. Klingaman, Ruby Leung, Jian Lu, Brian Mapes, Elizabeth A. Maroon, Sonali McDermid, Jong-yeon Park, Romain Roehrig, Brian E. J. Rose, Gary L. Russell, Jeongbin Seo, Thomas Toniazzo, Ho-Hsuan Wei, Masakazu Yoshimori, Lucas R. Vargas Zeppetello

**Affiliations:** 1 Institute of Meteorology and Climate Research - Department Troposphere Research, Karlsruhe Institute of Technology, Karlsruhe, Germany,; 2 Lamont-Doherty Earth Observatory, Columbia University, New York, New York, USA,; 3 Max Planck Institute for Meteorology, Hamburg, Germany,; 4 California Institute of Technology, Pasadena, California, USA,; 5 Sorbonne Universités, UPMC Univ Paris 06, Laboratoire d’Océanographie et du Climat, Paris, France,; 6 University of Wisconsin-Madison, Madison, Wisconsin, USA,; 7 Center for Climate Systems Research, Columbia University, New York, New York, USA,; 8 School of Urban and Environmental Engineering, Ulsan National Institute of Science and Technology, Ulsan, South Korea,; 9 National Centre for Atmospheric Science-Climate and Department of Meteorology, University of Reading, Reading, UK,; 10 Pacific Northwest National Laboratory, Richland, Washington, USA,; 11 Rosenstiel School of Marine and Atmospheric Sciences, University of Miami, Miami, Florida, USA,; 12 Department of Atmospheric Sciences, University of Washington, Seattle, Washington, USA,; 13 New York University, New York, New York, USA,; 14 Centre National de Recherches Météorologiques, UMR 3589, Meteo-France/CNRS, Toulouse, France,; 15 University at Albany (State University of New York), Albany, New York, USA,; 16 NASA Goddard Institute for Space Studies, New York, New York, USA,; 17 Uni Research, Bjerknes Centre for Climate Research, Bergen, Norway,; 18 Faculty of Environmental Earth Science and Arctic Research Center, Hokkaido University, Sapporo, Japan

## Abstract

This paper introduces the Tropical Rain belts with an Annual cycle and a Continent Model Inter-comparison Project (TRACMIP). TRACMIP studies the dynamics of tropical rain belts and their response to past and future radiative forcings through simulations with 13 comprehensive and one simplified atmosphere models coupled to a slab ocean and driven by seasonally varying insolation. Five idealized experiments, two with an aquaplanet setup and three with a setup with an idealized tropical continent, fill the space between prescribed-SST aquaplanet simulations and realistic simulations provided by CMIP5/6. The simulations reproduce key features of present-day climate and expected future climate change, including an annual-mean intertropical convergence zone (ITCZ) that is located north of the equator and Hadley cells and eddy-driven jets that are similar to present-day climate. Quadrupling CO_2_ leads to a northward ITCZ shift and preferential warming in Northern high latitudes. The simulations show interesting CO_2_-induced changes in the seasonal excursion of the ITCZ and indicate a possible state dependence of climate sensitivity. The inclusion of an idealized continent modulates both the control climate and the response to increased CO_2_; for example, it reduces the northward ITCZ shift associated with warming and, in some models, climate sensitivity. In response to eccentricity-driven seasonal insolation changes, seasonal changes in oceanic rainfall are best characterized as a meridional dipole, while seasonal continental rainfall changes tend to be symmetric about the equator. This survey illustrates TRACMIP’s potential to engender a deeper understanding of global and regional climate and to address questions on past and future climate change.

## 1. Introduction

The simulation of tropical rainfall is one of the most stubborn challenges in climate science. Despite general improvements, climate models still exhibit large-scale tropical rainfall biases such as a double intertropical convergence zone (ITCZ) in the Central-East Pacific that have now persisted more than two decades [*Mechoso et al*., 1995; *Hwang and Frierson*, 2013], and projections for both the ITCZ position [*Frierson and Hwang*, 2012; *Donohoe and Voigt*, 2016] and continental rainfall [*Knutti and Sedlacek*, 2013] remain uncertain in magnitude and sign. In many regions the models disagree (e.g., in the Sahel [*Biasutti et al*., 2008; *Park et al*., 2015]), and even in regions where they agree models are often at odds with recent trends (e.g., in East Africa [*Williams and Funk*, 2011; *Lyon and DeWitt*, 2012]). The uncertainty in projections of tropical rainfall stems mostly from uncertain changes in circulation patterns [e.g., *Chadwick et al*., 2013; *Xie et al*., 2015]. The response of circulation patterns to climate change arises from complex interactions and feedbacks between the large-scale flow, convection, and clouds that are not understood well enough to allow feasible rainfall projections. This gap in understanding has motivated the World Climate Research Program to establish a Grand Challenge on “Clouds, Circulation and Climate sensitivity” [*Bony et al*., 2015].

This paper introduces a hierarchical modeling approach that we have developed over the past 2 years to help answering the Grand Challenge question of “What controls the tropical rain belts?.” The “Tropical Rain belts with an Annual cycle and a Continent-Model Intercomparison Project” (TRACMIP) is a simulation suite that complements other modeling efforts [*Eyring et al*., 2015; *Webb et al*., 2016; *Zhou et al*., 2016] and has been performed by 13 comprehensive global climate models and 1 simplified gray-atmosphere model. The suite includes five simulations (see section 2 for details), two in aquaplanet configuration (prefixed Aqua) and three with an idealized tropical continent (prefixed Land). The Aqua- and LandControl simulations have a circular orbit and preindustrial greenhouse gas concentrations, while other experiments simulate the response to enhanced atmospheric carbon dioxide and changes in seasonal insolation. In all simulations, the atmosphere is thermodynamically coupled to a motionless slab ocean of uniform depth.

We envision that the design of TRACMIP and different pairings of the five simulations will shed light on different aspects of the dynamics of tropical rainfall and, more generally, the global climate. TRACMIP represents land as a rectangular patch of a very thin slab ocean with reduced evaporation and increased albedo. Soil moisture dynamics are thus disallowed, as are complications arising from continental geometry and the presence of topography and vegetation. This will allow us to identify which aspects of monsoonal circulations can be captured and understood with such a maximally idealized land, and which require more realistic land features [*Chou et al*., 2001]. Comparing Aqua and Land simulations can illuminate whether zonal asymmetries created by continental landmasses fundamentally change the behavior of the zonal-mean ITCZ in the control climates or in its response to greenhouse gas forcing. The juxtaposition of aquaplanet and idealized land simulations can further help us assess the extent to which established zonal-mean ITCZ frameworks provide useful information about regional rainfall characteristics [*Adam et al*., 2016a, 2016b], to what extent zonal asymmetries are required for monsoons to exist [*Bordoni and Schneider*, 2008], and how the presence of land modulates rainfall both locally and in the zonal-mean [*Maroon et al*., 2016]. The land simulations provide an update to the seminal work of *Chou et al*. [2001] and *Chou and Neelin* [2001, 2003] to investigate the importance of fully resolving the vertical structure of tropical circulations, interactions between tropical and extratropical circulations, and the representation of convection. Moreover, simulating the response to increased greenhouse gases and the response to seasonal insolation within the same model setups provides the foundation to build theories that encompass the key forcings of both future and past changes. This approach is similar to what has informed the design of the paleoclimate contribution to CMIP5 [*Schmidt et al*., 2014a] and will allow us to study to what extent and under which circumstances a theory built from past changes, e.g., the greening of the Sahara during the mid-Holocene, can inform and possibly constrain future changes [*Harrison et al*., 2015]. TRACMIP can thus fill the gap between work on past climate that used idealized models [*Merlis et al*., 2013a] and the comprehensive-model work done within the Paleo Model Intercomparison Project.

Model setups with idealized boundary conditions have become an important tool in the development of climate models and the investigation of climate dynamics [e.g., *Kang et al*., 2008; *Williamson et al*., 2012; *Stevens and Bony*, 2013; *Leung et al*., 2013; *Medeiros et al*., 2015; *Voigt et al*., 2014a; *Shaw et al*., 2015], and are now included in CMIP activities. Notably, CMIP5 included aquaplanet simulations with prescribed time-constant SSTs (forced with an approximation to current annual-mean SST and with a 4 K uniform warming) that build upon the AquaPlanet Experiment [*Williamson et al*., 2012] and were partly motivated by the Cloud Feedback Model Intercomparison Project. The CMIP5 aquaplanet simulations illustrate the large impact of moist processes on the atmospheric circulation and our gaps in understanding this impact [*Stevens and Bony*, 2013; *Voigt and Shaw*, 2015].

However, the CMIP5 use of fixed SSTs and the lack of seasonality might overemphasize model uncertainties that are less relevant for the dynamics of tropical rainfall in coupled realistic setups. When cloud and convective processes have full reign over the tropical rain belts, seemingly small changes in the convection scheme can lead to large changes in tropical rainfall [e.g., *Hess et al*., 1993; *Williamson et al*., 2012; *Moebis and Stevens*, 2012]. Yet when SST interactions and seasonality are present, convection is more constrained.

**Figure 1.**
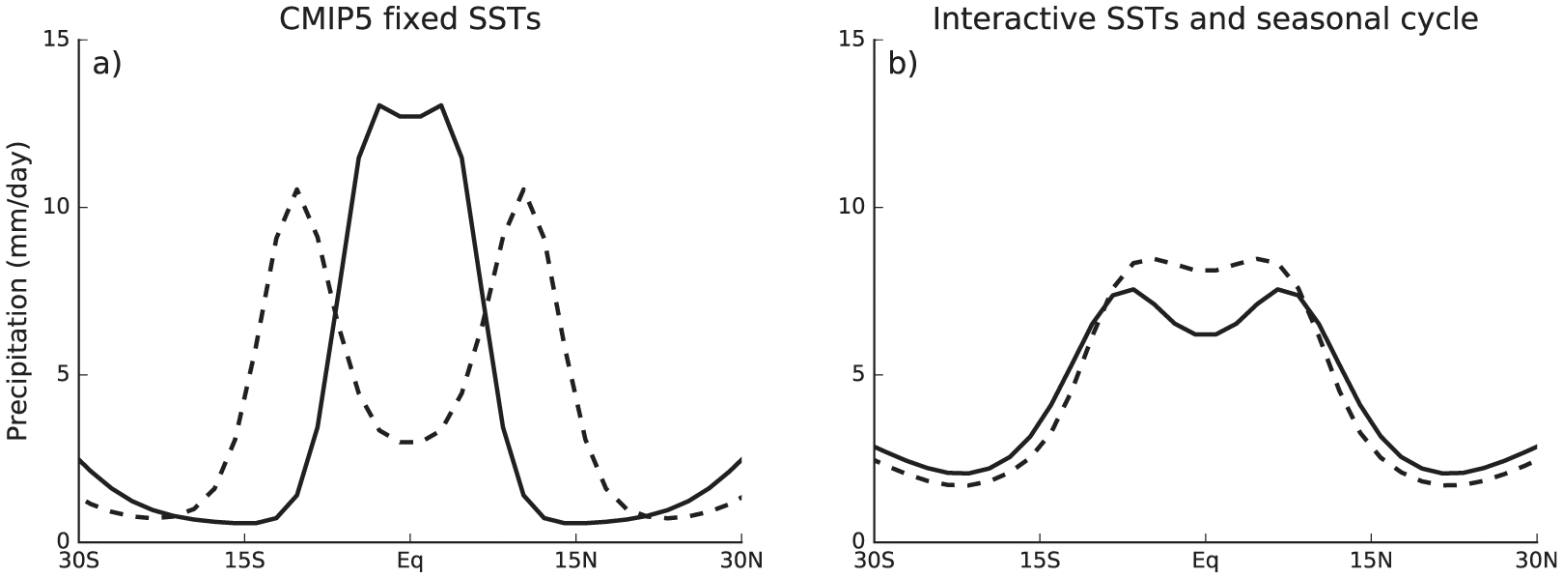
Equilibrium zonal-mean tropical precipitation in two different aquaplanet setups and two versions of the ECHAM6.1 atmosphere general circulation model. (a) CMIP5 aquaplanet setup with fixed SSTs and no seasonal cycle. (b) Aquaplanet setup coupled to a slab ocean with a seasonal cycle. The model versions only differ in their representation of moist convection (Nordeng convection for solid line, Tiedtke convection for dashed line; see *Moebis and Stevens* [2012] for details). While this difference has a large impact for fixed SSTs, its impact is strongly muted for interactive SSTs and a seasonal cycle. The simulations in Figure 1a use the CMIP5 aquaplanet setup [*Medeiros et al*., 2015], those in Figure 1b the setup of *Voigt et al*. [2014a, 2014b] with a slab ocean depth of 30 m and a peak ocean heat transport of about 2 PW in the subtropics.

An example is given in Figure 1 which shows tropical precipitation simulated by two versions of the ECHAM6.1 model (the atmospheric component of CMIP5 MPI-ESM Earth system model). The two versions only differ in the entrainment/detrainment rate of moist convection, but this small change is sufficient to create a stark difference in tropical precipitation between the two versions used in the CMIP5 aquaplanet setup, with one version simulating a single and the other version simulating a double ITCZ. Yet when used in an aquaplanet setup with interactive SSTs and a seasonal cycle, the precipitation differences largely vanish and both versions simulate similar ITCZs. This suggests that the ability of SSTs to respond to air-sea fluxes, including cloud-radiative effects, as well as the external timescale and the interhemispheric asymmetries set by the seasonal cycle, provide an anchor to the tropical climate. This hypothesis is supported by the interactive-SST aquaplanet work of *Lee et al*. [2008], who found that all of the studied models simulate a single ITCZ when run with a slab ocean (no seasonal cycle was used in that work). Model differences in cloud and convective processes, which have a strong impact in uncoupled CMIP5 aquaplanet simulations, might thus have much less of an impact in realistic coupled CMIP5 simulations. This raises the question to what extent the CMIP5 aquaplanet simulations are helpful to understand the model behavior in more realistic setups, and points to a gap in the model hierarchy provided by CMIP5. TRACMIP’s AquaControl simulation strives to fill this gap by using an interactive slab ocean and seasonally varying insolation. The aquaplanet simulations with quadrupled CO_2_ extend the bridge provided by TRACMIP between CMIP5 aquaplanets and realistic simulations to the case of future scenarios.

Importantly, TRACMIP fills this gap in the CMIP5 hierarchy not with a single model, but with an ensemble of models. MIPs, or the intercomparison of simulations performed by different climate models under identical boundary conditions, have revolutionized climate science. In particular, they have given researchers another method (complementary to single-model sensitivity experiments) to identify the processes that determine the response of a climate variable to external forcing. A model response that is consistent across an ensemble of GCMs carries more weight than results from a single model and is welcomed for that reason. But scatter across models can be just as informative. For example, correlations of anomalies across an ensemble can highlight how changes in two variables are connected to each other in a robust way across all models, even though the magnitude or even the sign of the changes are uncertain; these robust correlations point to robust mechanisms [e.g., *Biasutti et al*., 2009]. In particular, model intercomparisons identified “emergent constraints”: relationships that hold for both natural variability and anthropogenic changes and that, therefore, can be evaluated in observations of the former and used to constrain the latter [e.g., *Hall and Qu*, 2006; *Sherwood et al*., 2014]. Such constraints can be specific to a world region if the simulations are fully realistic, but they can also be specific to dynamical regimes and thus can also be identified in idealized model setups that add the advantage of a clean experiment [e.g., *Voigt et al*., 2014a; *Medeiros et al*., 2015].

Most models that contribute to TRACMIP are comprehensive global climate models. TRACMIP also includes an idealized model that represents convection in a simplified manner and that does not take into account radiative interactions of clouds and water vapor. The idealized model provides a link to past theoretical studies of tropical rain belt dynamics [*Chou and Neelin*, 2004; *Bordoni and Schneider*, 2008; *Kang et al*., 2009; *Merlis et al*., 2013b; *Bischoff and Schneider*, 2014]. We hope that this will foster TRACMIP’s aim to understand tropical rainfall dynamics across a hierarchy of models and boundary conditions and to better connect theories, state-of-the-art models, and ultimately observations [*Held*, 2005, 2014].

In this paper we introduce TRACMIP to the scientific community. First, we describe the experimental protocol, available diagnostics and participating models (section 2). The main part of the paper presents an overview of the mean climate simulated in the five configurations and highlights interesting aspects of the mean climate and its response in rainfall and temperature to external forcings: the control simulations without and with land are characterized in section 3 and the response to radiative forcing from CO_2_ and insolation changes is discussed in section 4. The full breadth and depth of the scientific inquiries that can be based on this data set is beyond the scope of any one paper and we will not attempt in our discussion (section 5) to fully answer any of the big-picture questions that TRACMIP was designed to address. Instead, we will discuss how TRACMIP can be used not just to investigate how tropical rain belts respond to climate change, but for a broad range of other purposes, from high-frequency tropical variability, to tropical-extratropical interactions and extratropical stormtracks. TRACMIP has been a community effort, and we are proud to share it as a community tool.

## 2. Design of TRACMIP

### 2.1. Experimental Protocol

**Figure 2.**
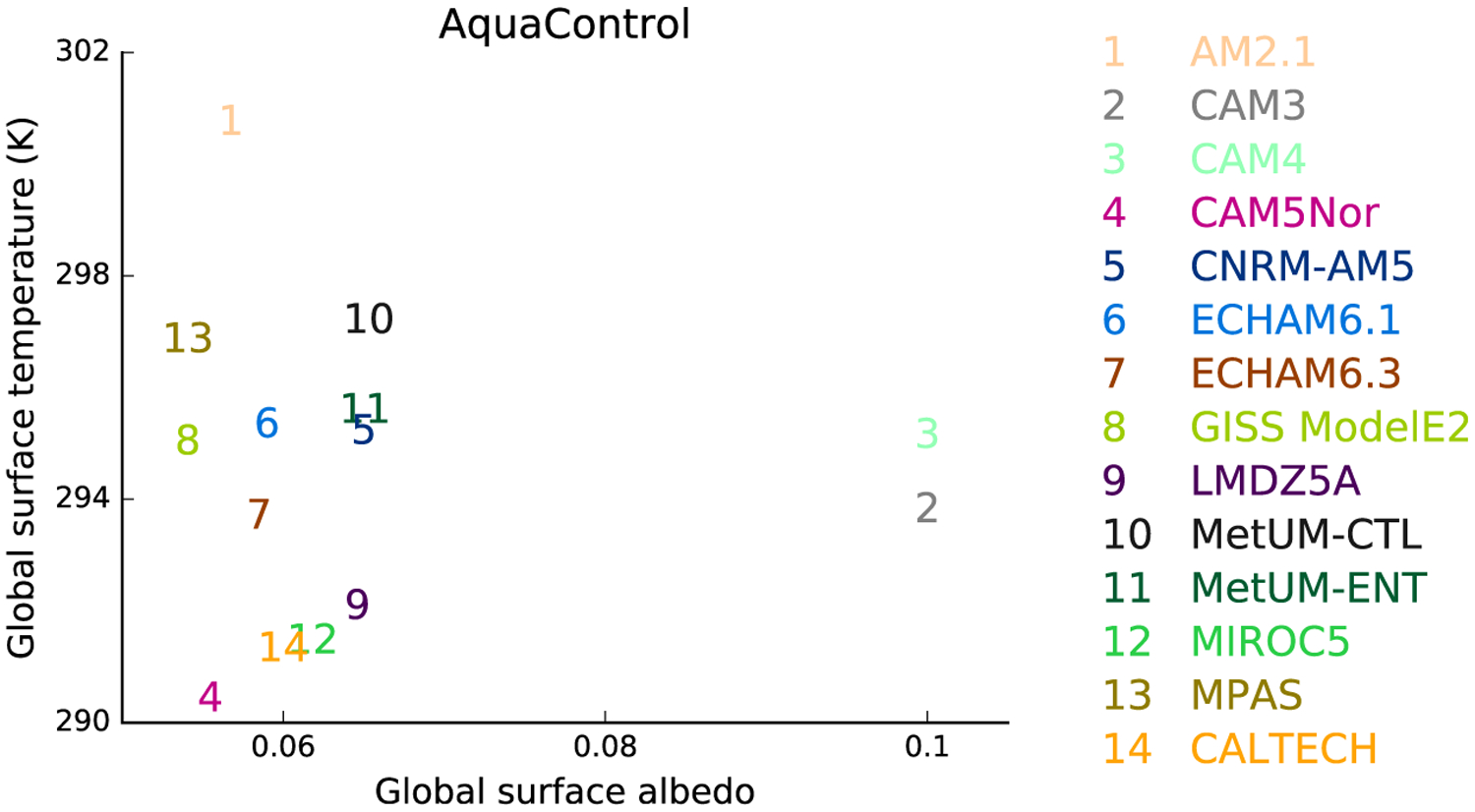
Global surface albedo and surface temperature in the AquaControl experiment. The surface albedo is calculated as the ratio of the global and time mean upward and downward shortwave radiative fluxes at the surface.

TRACMIP consists of five experiments that are listed in Table 1. The control experiment is an aquaplanet climate called AquaControl with zonally uniform boundary conditions. Aquaplanets have been employed previously, including in CMIP5, but in contrast to CMIP5 we couple the models to a thermodynamic slab ocean to close the surface energy balance and to allow for interactive sea-surface temperatures. A similar setup was proposed by *Lee et al*. [2008] and used in a small intercomparison by *Rose et al*. [2014], but here we also include a fixed northward meridional ocean heat transport and a seasonal cycle. Following the CMIP5 aquaplanet setup, greenhouse gases (with the exception of CFCs) and total solar irradiance are adapted from the AquaPlanet Experiment (APE) [*Williamson et al*., 2012]. AquaControl is forced by present-day CO_2_ = 348 ppmv, CH_4_ = 1650 ppbv, N_2_O = 306 ppbv, and a total solar irradiance of 1365 W m^−2^. Direct radiative effects of aerosols are set to zero, as are CFCs. Ozone is taken from APE (http://www.met.reading.ac.uk/~mike/APE/ape_ozone.html). Unlike in APE and CMIP5, physical constants such as gravitational acceleration and global-mean surface pressure are not specified, but the effect of model differences in these quantities is deemed negligible. TRACMIP includes the seasonal and diurnal cycles in insolation. With the exception of the LandOrbit experiment described below, the seasonal cycle is an idealized version of today’s insolation with an obliquity of 23.5° and zero eccentricity. The latter implies an annual-mean insolation that is symmetric with respect to the equator. Northern Hemisphere spring equinox is set to 21 March. The seasonal cycle enables seasonal north-south migrations of the ITCZ. To simulate seasonal ITCZ migrations comparable to today’s climate, the slab ocean depth is set to 30 m [*Donohoe et al*., 2014]. Modeling groups were asked to use a 360 day calendar, but since this was not available in all models some models use a 365 day calendar without (second option) or with leap years (third option). As in previous slab-ocean aquaplanet studies [e.g., *Kang et al*., 2008; *Voigt et al*., 2014a; *Rose et al*., 2014] sea-ice formation is turned off and the ocean is allowed to cool below the freezing temperature. Models use their own surface roughness length and ocean albedo. Model differences in ocean albedo do not appear to be the cause of model differences in global surface temperature (Figure 2), as Earth’s energy balance is more strongly controlled by atmospheric processes, in particular clouds [*Donohoe and Battisti*, 2011].

Four more experiments study the impact of CO_2_, land, and insolation. The first is an aquaplanet experiment initiated from AquaControl with CO_2_ instantaneously quadrupled and is called Aqua4xCO2. This experiment mimics the CMIP5 coupled Abrupt4xCO2 experiments and is designed to provide insights into the equilibrium response to the greenhouse gas forcing as well as its transient evolution. The lack of a dynamic ocean means, however, that the transient response in TRACMIP focuses on the mixed-layer response of the ocean on decadal timescales and does not account for the impacts of spatially and time-varying ocean heat uptake (see *Rose and Rayborn* [2016] for a recent review).

The other three experiments are performed with a modified lower boundary designed to capture the essential characteristics of a continent. The continent is a flat rectangular region that straddles the equator in a fashion analogous to the African continent, reaches into the subtropics (30°S–30°N), and is limited in longitude to a width of 0°E–45°E. Because the primary focus of TRACMIP is on atmospheric processes, we choose to avoid the complication of land surface schemes and soil moisture feedbacks, and implement a continent made neither of land nor of water—a “jello” continent. Land is modeled as a thin (0.1 m) slab of ocean with albedo increased by 0.07 compared to the models’ own ocean albedo, suppressed ocean heat transport (i.e., zero q-flux), and reduced evaporation. The reduction in evaporation is achieved by halving the surface exchange coefficient for moisture, *C*_*q*_, used in the calculation of the surface evaporative flux *E*,

(1) $$E=C_{q}v(q-q_{s}),$$

where *v* is a measure of near-surface wind speed, *q* is near-surface specific humidity, and *q*_*s*_ is the saturation-specific humidity for a given surface temperature. Over land, equation (1) is changed to

(2) $$E=\frac{1}{2}C_{q}v(q-q_{s}),$$

which, assuming changes in surface wind speed and boundary-layer humidity are small, will reduce evaporation by a factor of 2. While evaporation is always suppressed, land can never dry out in TRACMIP, in contrast to what would happen with a bucket model formulation [e.g., *Manabe*, 1969]. Over ocean equation (1) is applied in all experiments. The surface roughness is the same over land and ocean.

**Figure 3.**
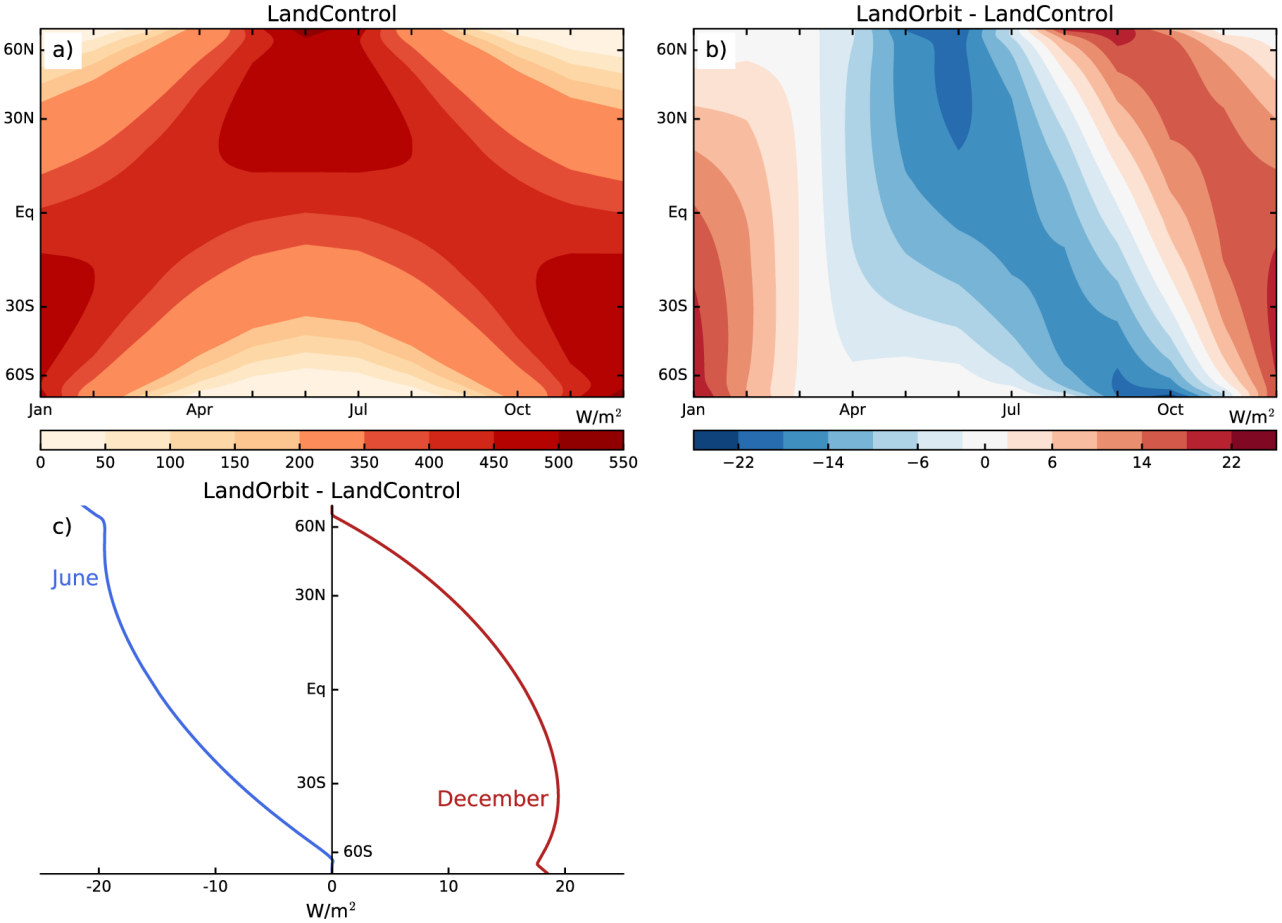
Model median seasonal insolation in (a) LandControl, (b) the difference between LandOrbit and LandControl, and (c) highlighted for the Northern and Southern Hemisphere summer months. Models slightly differ in the seasonal insolation changes because of different calendars, for which reason the model median is shown.

The three land experiments are LandControl, Land4xCO2, and LandOrbit. LandControl differs from AquaControl only by the introduction of the continent. Land4xCO2 and LandOrbit are initiated from LandControl and study the response to radiative forcing. Land4xCO2 has instantaneously quadrupled CO_2_. In LandOrbit a nonzero eccentricity of *ϵ*=0.02 is introduced to create a hemispheric difference in seasonal insolation such that compared to LandControl, Northern and Southern Hemisphere receive less and more insolation in their respective summers. Annual-mean insolation in LandOrbit is the same as in all other experiments; the seasonal insolation changes are shown in Figure 3. The eccentricity change addresses the seasonal insolation change due to precessional forcing that is responsible for the dominant signal in Holocene tropical hydroclimate [e.g., *Prell and Kutzbach*, 1987; *Clemens et al*., 2010]. The choice of comparing simulations with and without eccentricity, instead of simulations with the same eccentricity but different time of perihelion, was made in order to have the simplest possible control simulation (*ϵ* = 0), in which the only source of hemispheric asymmetry is the ocean heat flux (see below). The insolation in LandOrbit roughly corresponds to today’s orbit [*Joussaume and Braconnot*, 1997], so that the insolation difference of LandControl-LandOrbit is about half as strong as the insolation change between the mid-Holocene and today.

**Figure 4.**
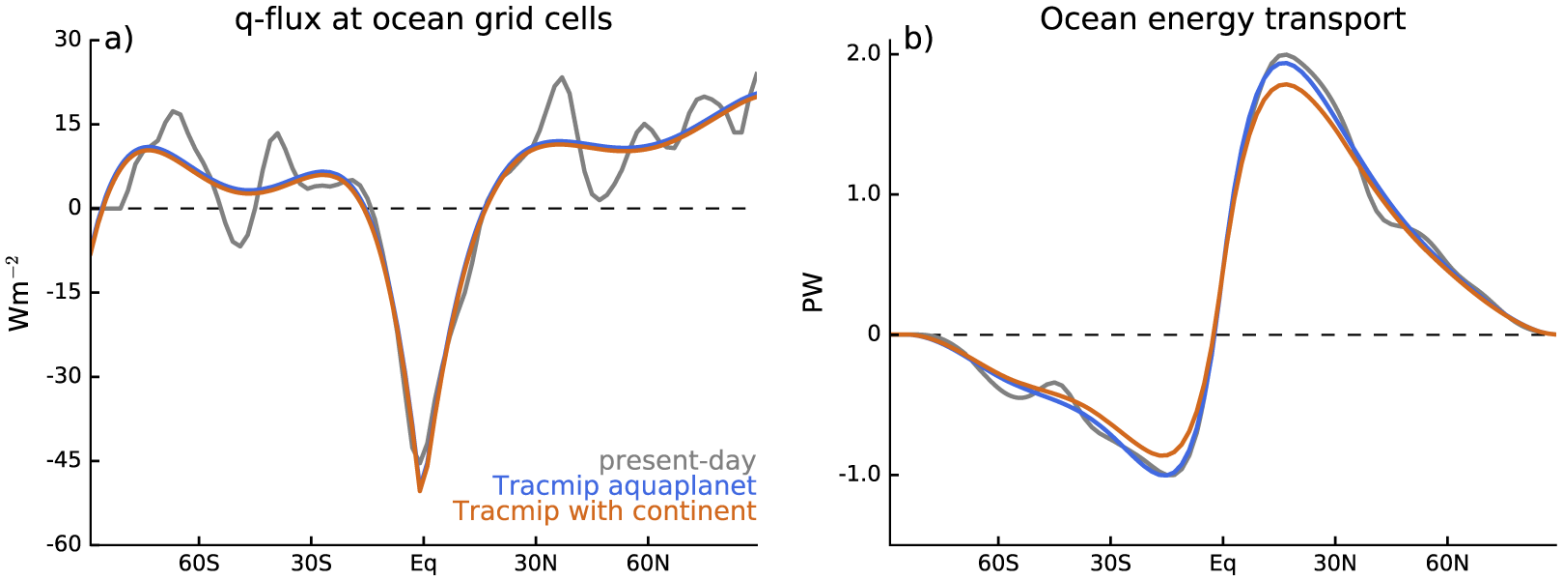
(a) q-flux over ocean grid boxes and (b) associated total meridional ocean heat transport. The TRACMIP q-flux is a fourth-order polynomial fit to the observed q-flux shown in gray. The q-flux for simulations with land is set to zero over land, which requires a small decrease of q-flux compared to the aquaplanet simulations to ensure that the global-mean q-flux is zero. As a result of replacing some of the tropical ocean grid boxes with land, the total ocean energy transport is slightly reduced in simulations with land compared to aquaplanet simulations.

The slab ocean includes a prescribed ocean heat transport that is imposed as a so-called “q-flux” in units of W m^−2^. The q-flux is added to the surface energy balance and cools low latitudes and warms mid and high latitudes, mimicking the effect of meridional energy transport of a dynamic ocean. The TRACMIP q-flux is zonally symmetric and constant in time. It is an approximation to the zonal and time mean q-flux of the present-day climate that is shown in Figure 4a and that we calculated from observations of top-of-atmosphere radiative fluxes from CERES and moist static energy divergence from the ERA-Interim reanalysis, both averaged over years 2001–2010 (see *Frierson et al*. [2013] for details). The zonal average includes land points for which the q-flux is set to zero. Small-scale meridional variability in the observed q-flux in mid and high latitudes arguably is impacted by the specific land-ocean geometry of the present-day Earth, and so for TRACMIP we meridionally smooth the q-flux by fitting a fourth-order polynomial to the observed q-flux,

(3) $$q(\varphi )=p_{0}+p_{1}\varphi +p_{2}\varphi^{2}+p_{3}\varphi^{3}+p_{4}\varphi^{4},$$

where *φ* is degree latitude. The fit is done separately for the Northern and Southern Hemisphere, leading to hemispherically dependent coefficients listed in Table 2. In the simulations with land, the q-flux is set to zero over land. This requires a small q-flux correction of −0.59 W m^−2^ over ocean points in the land simulations to ensure that the global-mean q-flux is still zero. The correction is applied to all ocean points as a small decrease in *p*_0_, which implies a small cooling over ocean in the land simulations compared to the aquaplanet simulations. The meridional energy transport in PW associated with the q-flux is shown in Figure 4b. At the equator the ocean transports 0.5 PW into the Northern Hemisphere. This is consistent with the present-day climate [*Ganachaud and Wunsch*, 2000; *Frierson et al*., 2013; *Marshall et al*., 2013] and puts the annual-mean ITCZ into the Northern hemisphere in TRACMIP, as is described in more detail in section 3.

### 2.2. Requested Diagnostics and Participating Models

While TRACMIP is not organized within CMIP6, partly because the idea for the project became evident only in the fall of 2014, TRACMIP attempts to leverage past and future CMIP activities as much as possible. Many of the contributing models are either CMIP5 models or recent developments that reflect preparations for CMIP6. Modeling groups were asked to prepare their data according to the CMIP5 conventions for variable names, units and signs (i.e., to “cmorize” the data). The requested fields are those specified in the CMIP5 atmospheric Amon table, which is available at http://cmip-pcmdi.llnl.gov/cmip5/docs/standard_output.pdf (excluding those related to the chemical composition of the atmosphere). Three-dimensional atmospheric data are interpolated on the 17 CMIP5 pressure levels (1000, 925, 850, 700, 600, 500, 400, 300, 250, 200, 150, 100, 70, 50, 30, 20, and 10 hPa). The fields are requested as monthly, daily and 3 h data to enable studies that connect the models’ climatologies to fast processes on daily and subdaily timescales. TRACMIP also follows CMIP5 regarding whether fields should be saved as averages or snapshots. For the monthly and daily output streams, all fields are requested as averages over the daily or monthly output period. For the 3 h output stream, surface and atmospheric temperature, horizontal wind, vertical wind, specific humidity, and geopotential height are requested as snapshots, and all other fields as averages. For each experiment, monthly output is requested for all years (except the 15 years of spin-up in AquaControl; for all models global-mean surface temperature has equilibrated at year 15 of AquaControl), daily output for the last 10 years, and 3 h output for the last 3 years. To enable studies of the transient response, all experiments except AquaControl are restarted from another experiment as described in Table 1.

TRACMIP was very well received by the scientific community. So far 13 comprehensive climate models and one simplified climate model have contributed simulations (see Table 3). With a few exceptions, almost all models have performed all experiments. This can be read off from the summary of global-mean time-mean surface temperature and time-mean ITCZ position given in Table 4 for each model and experiment. Some of the 13 comprehensive models only differ in specifics of the physical parameterizations, allowing for a judgment of how changes in the treatment of clouds and convection impact tropical rain belts. For example, the MetUM model is run in two configurations CTL and ENT that differ in the parameter settings for convection [see also *Klingaman and Woolnough*, 2014; *Bush et al*., 2015]. Similarly, ECHAM6 is run in two versions 6.1 and 6.3; and three different versions of the CAM Community Atmosphere Model are used. This judgment is further facilitated by the inclusion of the idealized model CALTECH that does not take into account radiative feedbacks from clouds and water vapor and represents moist convection in a simplified manner. The CALTECH model uses a gray radiation scheme, in which absorption and emission of solar and thermal radiation do not depend on wavelength. Hence, in this model an equivalent 4xCO_2_ experiment is run by increasing the prescribed longwave optical thickness in the gray scheme.

**Figure 5.**
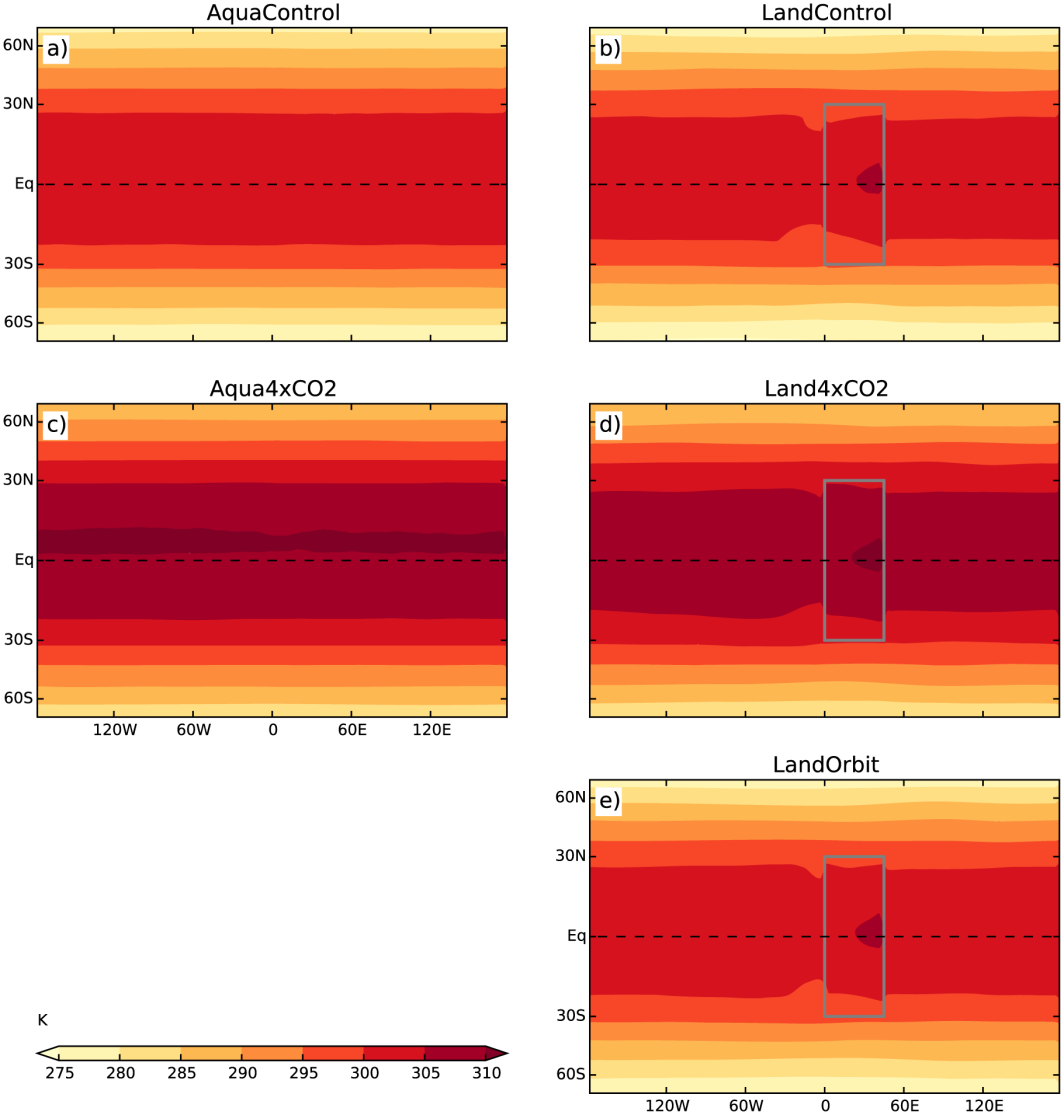
Model median of annual-mean surface temperature for the five TRACMIP experiments. The continental boundaries for the simulations with land (right column) are indicated by the gray rectangle.

**Figure 6.**
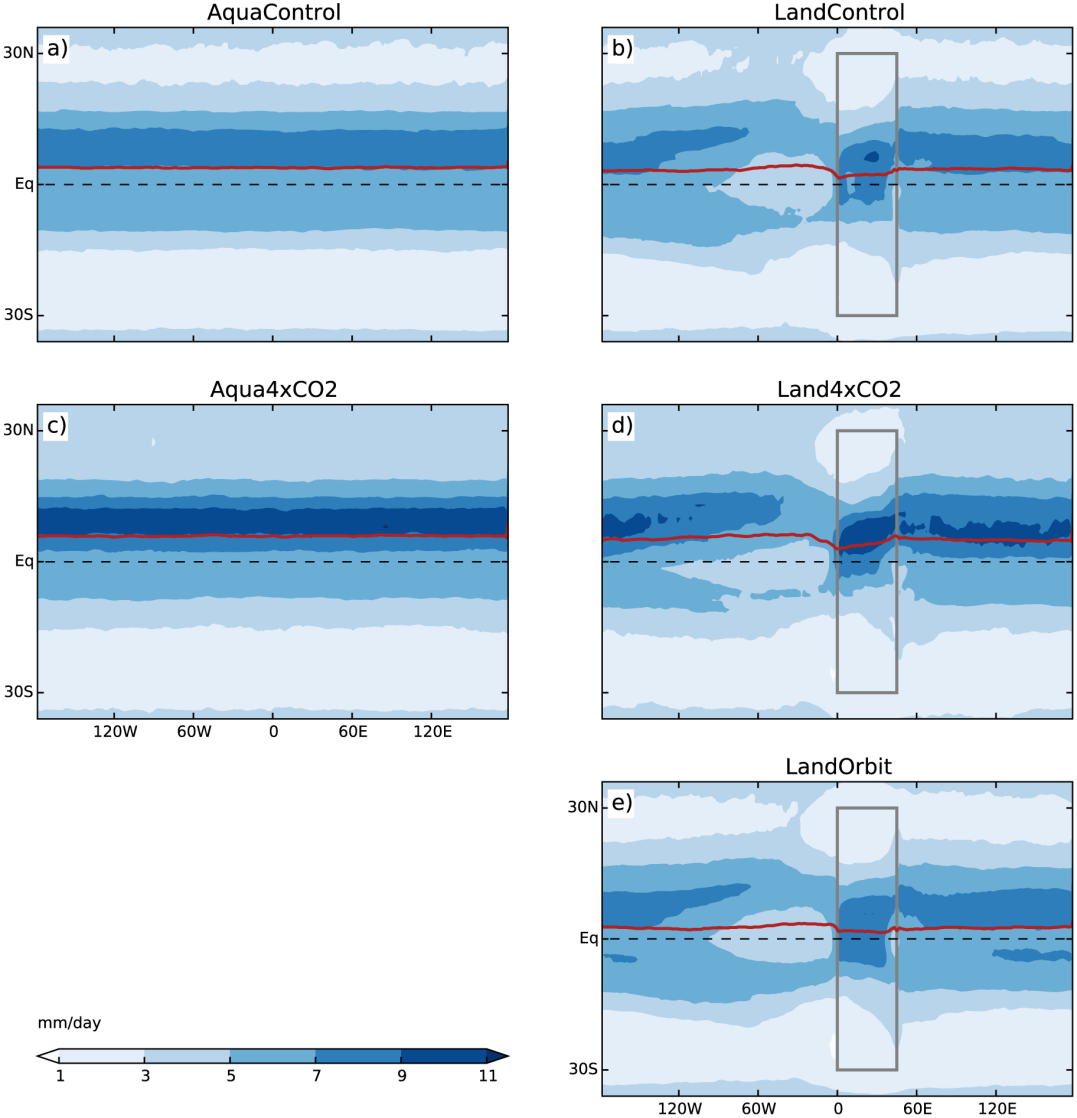
Model median of annual-mean precipitation for the five TRACMIP experiments. The continental boundaries for the simulations with land (right column) are indicated by the gray rectangle. The red line is the ITCZ position calculated as the latitude of the model median precipitation centroid between 30°N and 30°S.

**Figure 7.**
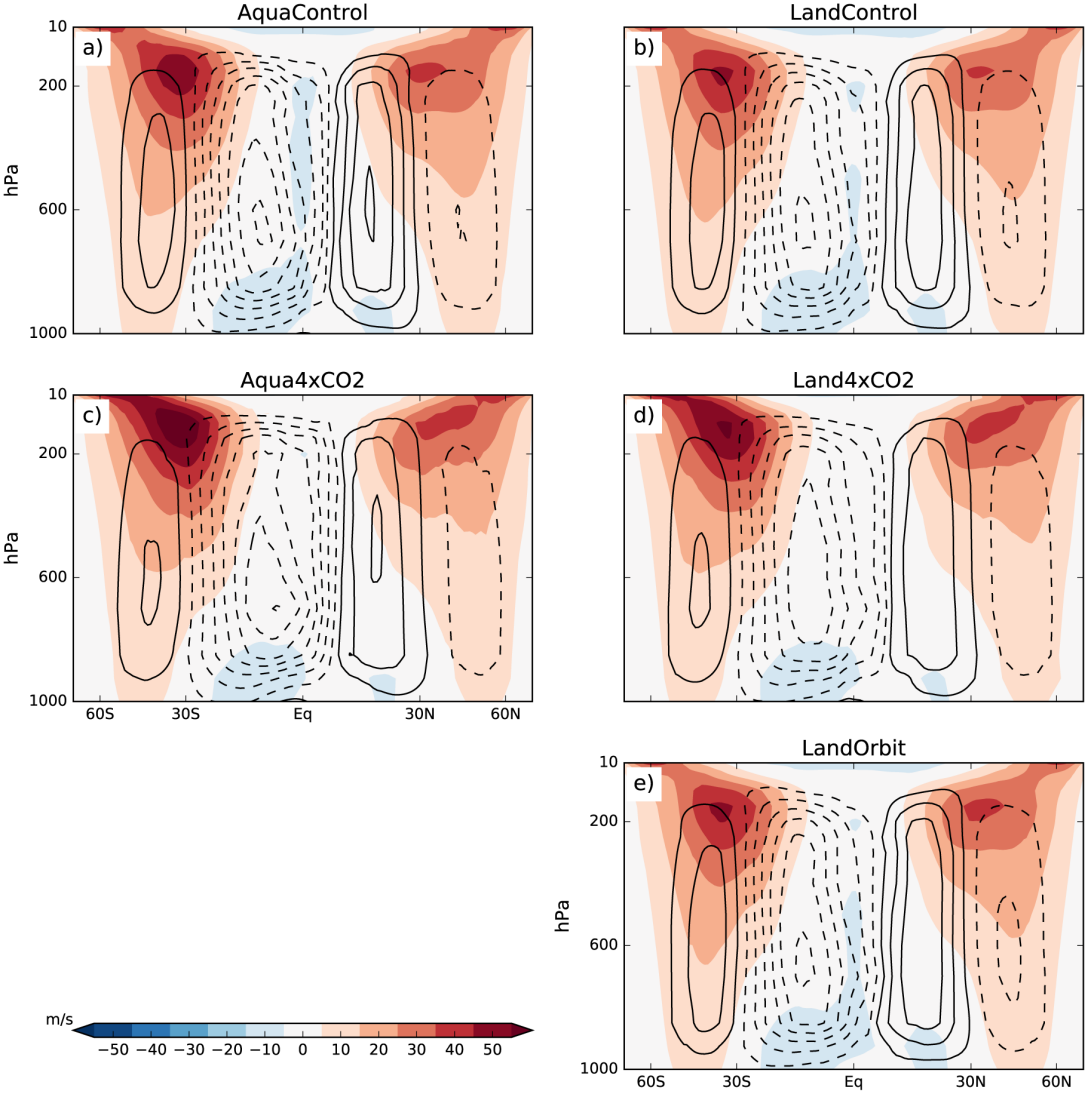
Model median of annual-mean zonal-mean zonal wind and mass stream function in the five TRACMIP experiments. The zonal wind is shown in colors and the mass stream function in contours. For the mass stream function, solid contours indicate clockwise flow, dashed contours indicate counterclockwise flow, and the contour interval is 20×10^9^ kg s^−1^.

For reference Figures 5–7 show the model median of annual-mean surface temperature, precipitation, zonal-mean zonal wind, and meridional mass stream function in all five TRACMIP experiments. Throughout this paper, the last 20 years of each simulation are analyzed and models are interpolated on a common 1° × 2° latitude-longitude grid for the calculation of the model median values.

## 3. Control Climate for a Zonally Symmetric Aquaplanet and a Tropical Continent

In this section we describe the control climates of the aquaplanet setup and the setup with land. We begin with AquaControl and then compare it to LandControl to characterize the global and local impact of land. Unless otherwise stated we discuss the annual-mean climate.

### 3.1. AquaControl

In the aquaplanet setup the models simulate an annual global-mean surface temperature of 290.4–300.7 K (model median of 295.1 K; Figure 2 and Table 4). The models are about 3–12 K warmer than the present-day climate, and warmer than realistic coupled CMIP5 simulations of the twentieth century. This is expected from the lack of sea ice and continental areas, which both have a higher albedo than ocean, as well as the lack of aerosol-radiative interactions.

Eight of the 14 models are within ±2 K of the model median. The model spread in TRACMIP global surface temperatures is higher than in historical CMIP5 simulations, which show a model spread of around 3 K [*Mauritsen et al*., 2012]. Yet the model spread in global surface temperature is still small enough to justify a meaningful comparison between the models, and is smaller than what one might have expected given that models were not tuned to a specific target temperature for TRACMIP, in contrast to CMIP5.

**Figure 8.**
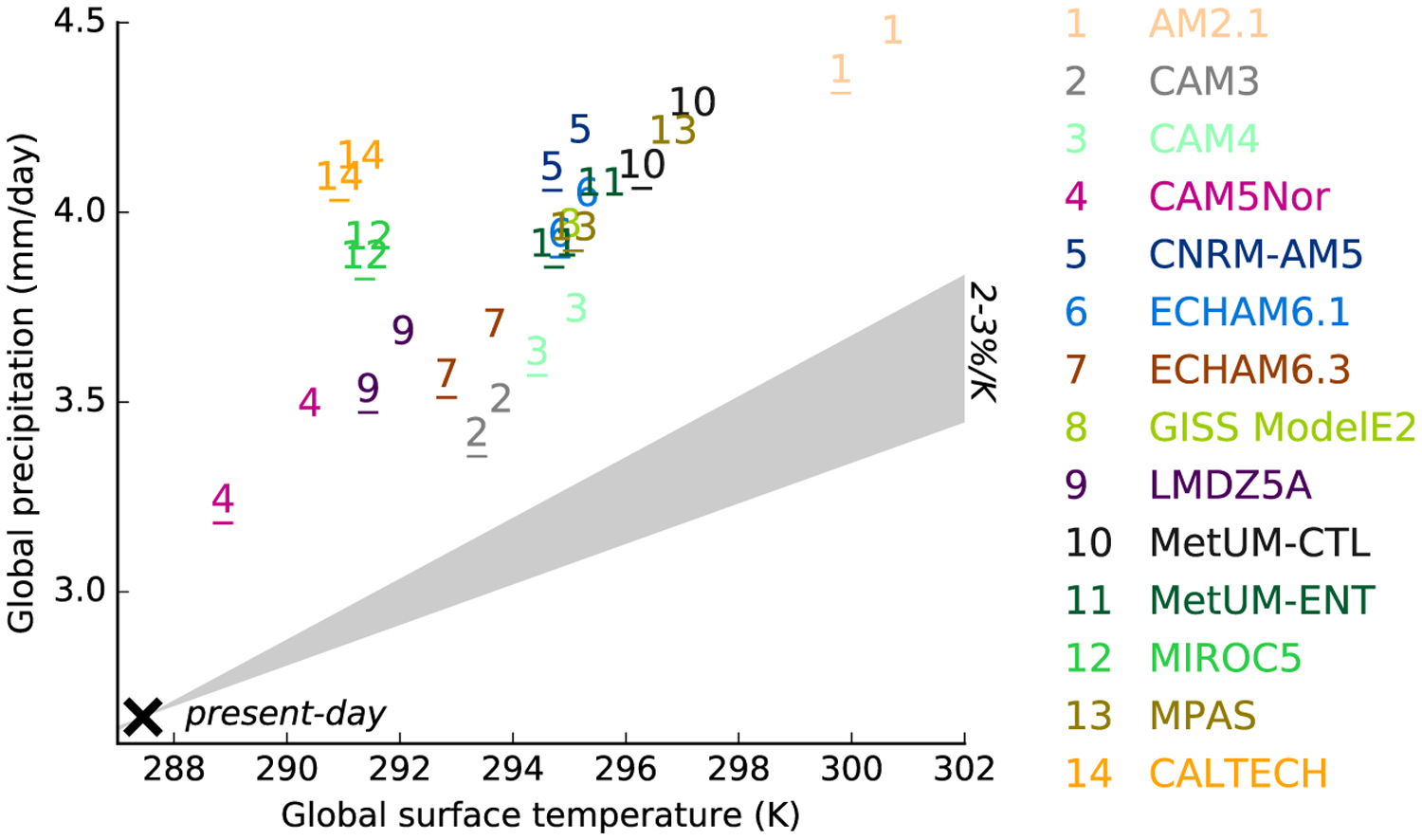
Global precipitation as function of global surface temperature in the AquaControl (no underscore) and LandControl (with underscore) experiments. The cross indicates the present-day (1979–2010) surface temperature of 14.3°C (taken from HadCRUT4) [*Morice et al*., 2012] and precipitation of 2.7 mm/d (taken from GPCPv2.2) [*Adler et al*., 2003]. The triangle is the extrapolation of present-day precipitation to warmer climates assuming a 2–3% precipitation increase per degree surface warming.

Figure 8 shows global precipitation as a function of global surface temperature. The models simulate a global-mean precipitation of 3.5–4.5 mm/d (model median of 4.0 mm/d). Precipitation is about 50% higher compared to present-day (2.7 mm/d GPCPv2.2; 1979–2010). This is only partly explained by the warmer climates in TRACMIP. When the present-day precipitation is extrapolated to the TRACMIP surface temperatures assuming a 2–3%/K precipitation scaling following *Held and Soden* [2006] (gray shading in Figure 8), TRACMIP precipitation is still larger. TRACMIP precipitation is higher not only because of a warmer climate but also because of the lack of continental areas, over which evaporation can be moisture limited and sensible heat fluxes play a larger role than over ocean.

**Figure 9.**
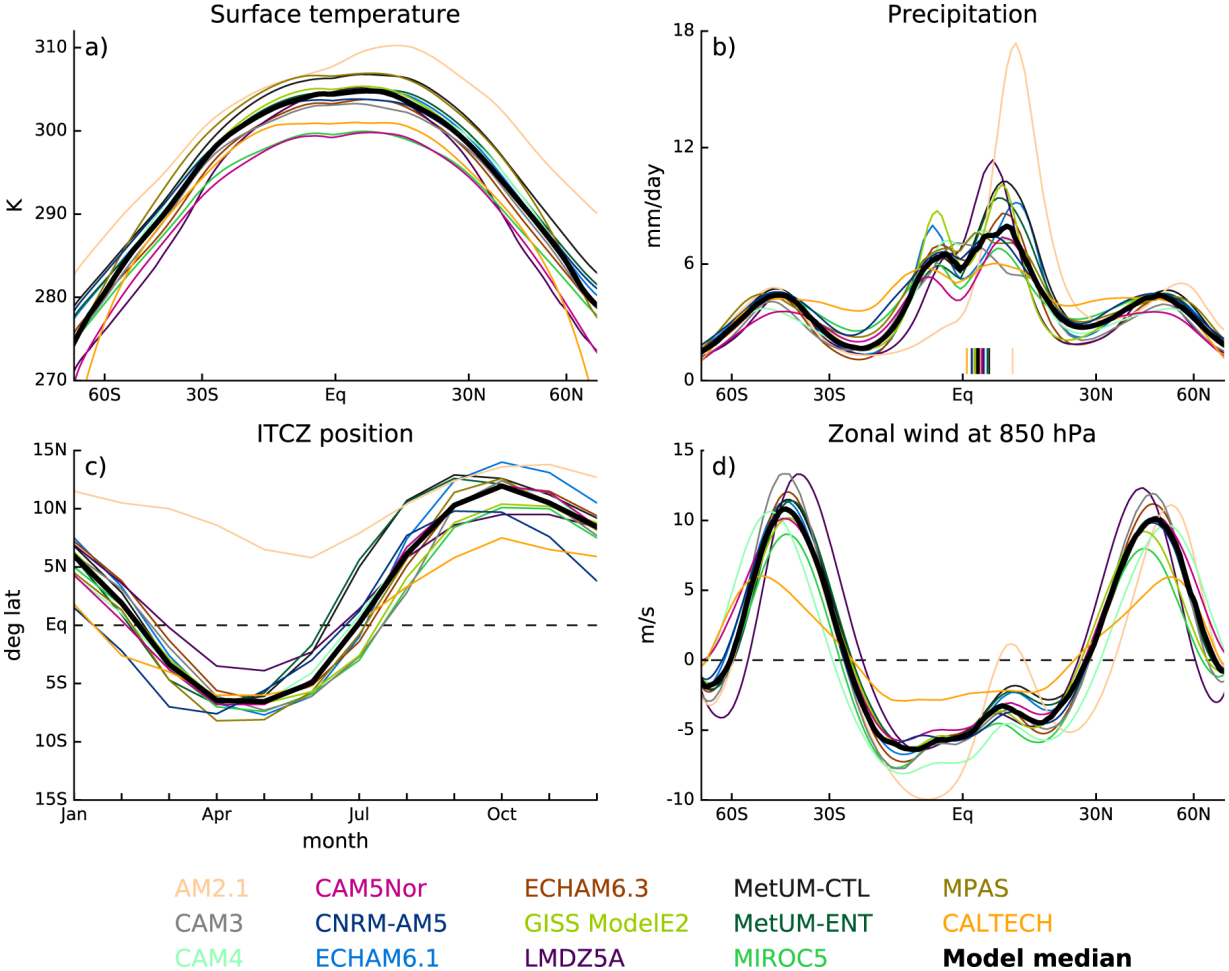
Zonal-mean characteristics of the AquaControl experiment. (a) Annual-mean surface temperature, (b) annual-mean precipitation, (c) seasonal evolution of the ITCZ position, and (d) annual-mean zonal wind at 850 hPa. Individual models are shown by the colored lines, the model median is shown by the thick black line. In Figure 9b the vertical lines show the annual-mean ITCZ position.

The ensemble-median annual mean patterns of surface temperature and precipitation of the AquaControl were shown in Figures 5a and 6a; as expected the climate is zonally symmetric aside from very small residual noise. The zonal-mean median and all individual models are shown in more compact form in Figure 9, which shows annual-mean temperature, precipitation, and lower tropospheric zonal wind (see also Figure 7) as well as the seasonal progression of the ITCZ. Following *Frierson and Hwang* [2012], the ITCZ is defined as the latitude of the precipitation centroid between 30°N and 30°S (same area-integrated annual-mean precipitation north and south of the ITCZ).

The Northern Hemisphere is 0.9–5.3 K (model median 2.2 K) warmer than the Southern Hemisphere in the hemispheric mean, and is also warmer at all corresponding latitudes (Figure 9a). The warmer Northern Hemisphere is consistent with northward cross-equatorial ocean heat transport, which has been invoked to explain the 1–2 K warmer Northern Hemisphere of the present-day climate [*Feulner et al*., 2013; *Kang et al*., 2015]. However, since the hemispheric difference in TRACMIP is much larger than in the present-day climate even though the ocean transports the same amount of energy across the equator, other processes, likely those involving radiative interactions of clouds and water vapor, must play a role as well. The meridional profile of surface temperature is rather different from the present-day climate, as the lack of land, ice, and mountains strongly reduces the equator to pole contrasts. For example, the surface temperature contrast across the Southern Hemisphere is about 30 K, roughly half of that in the real world.

The annual-mean tropical precipitation has a double peak structure, but the Northern Hemisphere peak is dominant, so that the annual-mean ITCZ position, defined as the precipitation centroid, is at 0.9°N–10.8°N (model median 3.3°N) (Figure 9b and Table 4). This is consistent with the northward cross-equatorial ocean energy transport [*Kang et al*., 2008; *Frierson et al*., 2013; *Marshall et al*., 2013]. Over the course of the seasonal cycle, the ITCZ migrates back and forth across the equator (except for AM2.1), reaching its most northern excursion in October and its most southern excursion around April–May (Figure 9c); this indicates a seasonal cycle that is lagged behind the real world, where the presence of land mitigates the longer response time of the mixed-layer ocean [*Biasutti et al*., 2004]. The specific choice of the mixed-layer depth has, of course, a large impact on the exact timing of the seasonal peak. During most months tropical precipitation has only one peak (not shown), implying that the double peak in tropical annual-mean precipitation is the result of the seasonal migrations of a single ITCZ. The annual-mean circulation shows a Northern Hadley cell of 42–99 × 10^9^ kg s^−1^ (model median 72 × 10^9^ kg s^−1^) and a Southern Hadley cell of −53 to −265 × 10^9^ kg s^−1^ (model median −133 × 10^9^ kg s^−1^). The eddy-driven jet, defined as the 850 hPa zonal wind maximum [*Barnes and Polvani*, 2013], is at 42°N–51°N (model median 46°N) in the Northern Hemisphere and at 38°S–49°S (model median 42°S) in the Southern Hemisphere (Figure 9d).

Overall, TRACMIP’s AquaControl reproduces the main features of the present-day climate: the Northern Hemisphere is warmer than the Southern Hemisphere, the ITCZ is located in the Northern Hemisphere in the annual mean and migrates back and forth across the equator, the annual-mean Hadley circulation is stronger in the Southern Hemisphere than in the Northern Hemisphere, and the eddy-driven jets are located at around 45°N/S. The TRACMIP AquaControl simulations are thereby closer to the present-day climate than the CMIP5 prescribed-SST aquaplanet simulations, which show an excessively strong Hadley circulation, too equatorward eddy-driven jets, and no hemispheric asymmetry [*Medeiros et al*., 2015].

### 3.2. LandControl

We now describe the climate impact of the tropical “jello” continent by comparing LandControl and AquaControl. Introducing land leads to a global-mean cooling of −0.1 to −1.8 K (model median −0.7 K) and a precipitation decrease of −0.05 to −0.25 mm/d (model median −0.11 mm/d) (Figure 8 and Table 4). This global cooling might be expected if one assumed that the land-induced increase in surface albedo translated to a similar change in planetary albedo. However, a closer look at the pattern of surface temperature change indicates that matters are more complicated.

**Figure 10.**
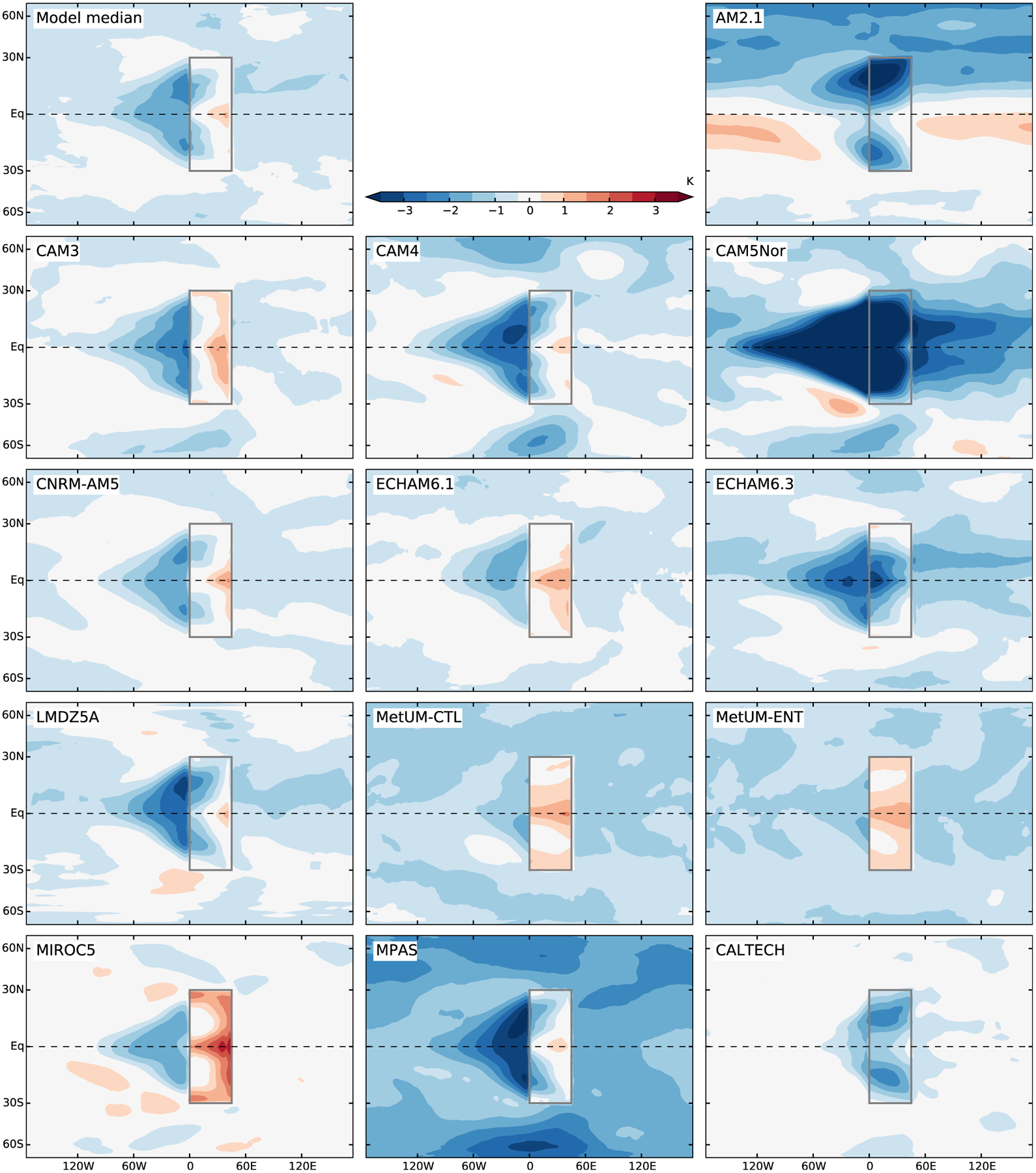
Impact of the tropical continent on surface temperature: annual-mean surface temperature difference between LandControl and AquaControl. The continent is indicated by the gray box.

Figure 10 shows the annual mean change in surface temperature that results from introducing the tropical continent (LandControl-AquaControl) in the model median and in each model. In the model median the cooling does not predominantly arise from the change in surface temperatures over land but rather from the general cooling of the global ocean and the even stronger cooling in the region just west of the continent. The temperature change differs substantially in sign and magnitude between models, however. In some models the land warms with respect to the aquaplanet setup, while it cools in others. Many models show a wedge of ocean cooling west (i.e., downstream) of the continent that extends along the equator from the coast to between 60°W and 120°W, but the details of this feature are not robust across models. The introduction of land thus has a clear impact on regional temperatures, but this impact differs markedly between models. Much of the temperature pattern response to the introduction of land and the model difference therein appears to be mediated by clouds. This can be seen from the CALTECH model, in which clouds are missing, the cooling maximizes over land consistent with the local surface albedo increase, and the ocean cooling west of the continent that is seen in the comprehensive models is nearly absent.

**Figure 11.**
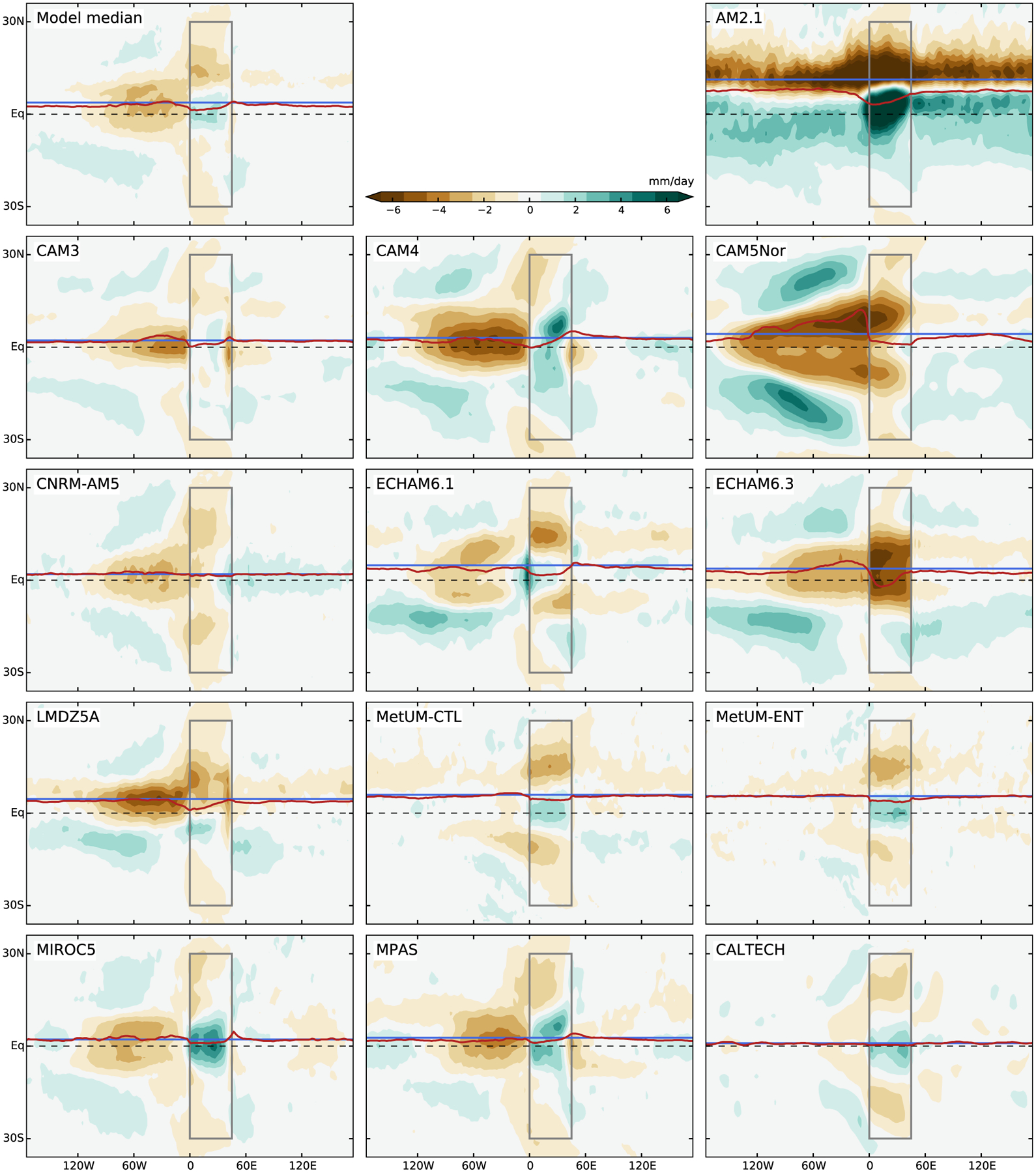
Impact of the tropical continent on precipitation: annual-mean precipitation temperature difference between LandControl and AquaControl. The continent is indicated by the gray box. To highlight the impact on tropical precipitation, the plot is restricted to latitudes between 40°N and 40°S. The blue and red lines show the location of the precipitation centroid (defined between 30°N/30°S) at every longitude in AquaControl and LandControl, respectively.

Figure 11 shows the annual mean change in low-latitude precipitation due to the tropical continent. The shading indicates the LandControl-AquaControl anomalies, while the colored lines indicate the precipitation centroid at each longitude in each experiment (blue for AquaControl and red for LandControl; see also Figure 6). In the model median, precipitation is increased near the equator over land but reduced over the Northern subtropical part of the continent and over the near-equatorial ocean west of the continent (creating an isolated global maximum of precipitation over land at about 5°N and away from the coast, see Figure 6). There is also a hint of increased precipitation over the subtropical ocean in the western hemisphere. As was the case for surface temperature, however, models differ markedly on the regional precipitation change, and the regional changes in the model median are not completely robust across models. For example, ECHAM6.3 dries the equatorial continent, while AM2.1 wets at all equatorial longitudes. The precipitation changes are associated with substantial changes in the ITCZ position; these are largest at the location of the continent, but are not limited to it and do not sum up to zero in the zonal mean. The zonal-mean, time-mean ITCZ shifts southward in LandControl compared to AquaControl in all models but one (0.0 to −4.2° lat; model median −0.6° lat; Table 4). This happens even though the continent is symmetric with respect to the equator, implying that the southward ITCZ shift must result from a rectification of hemispheric asymmetries in the atmospheric energy budget. The ITCZ shift varies zonally. All models simulate a southward ITCZ shift over land; this extends downstream over the ocean in some models, but in others the precipitation is shifted north for a span of 30–60° longitude. Overall, this shows that even a small continent that only covers 1/16th of Earth’s surface has a clear impact on tropical rain belts and causes important regional variations from the zonal mean. Future studies are needed to elucidate by which mechanism the median change is achieved and why some models behave as outliers in their temperature or rainfall responses.

## 4. Response to Radiative Forcing From Quadrupled CO_2_ and Seasonal Insolation Changes

In this section we characterize the response of the AquaControl and LandControl climates to increased CO_2_ and seasonal insolation changes. As in section 3 we focus on the annual-mean climate.

### 4.1. Response to Quadrupled CO_2_

**Figure 12.**
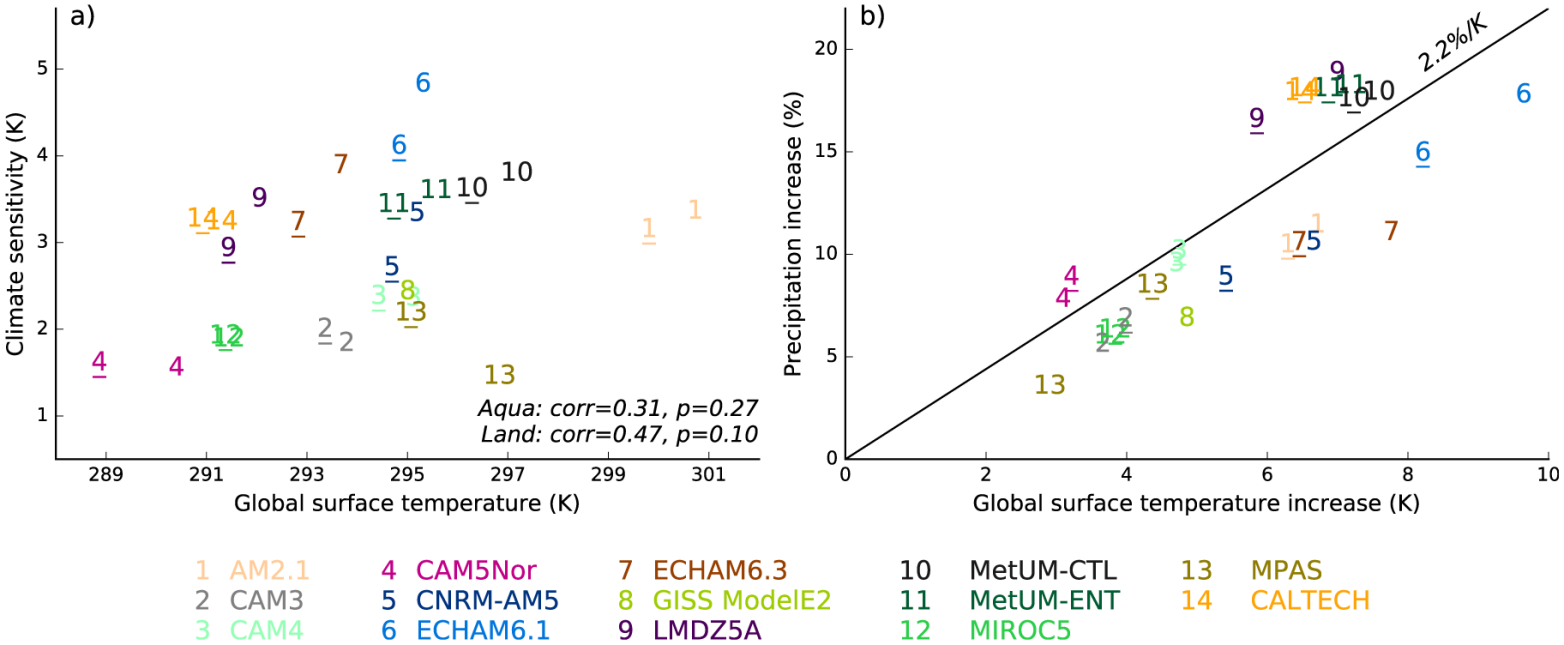
Climate sensitivity and hydrological sensitivity in the TRACMIP ensemble. (a) Climate sensitivity as estimated by halving the global surface temperature change between the Control and 4xCO2 experiments for aquaplanet simulations (no underscore) and land simulations (with underscore). The numbers give the correlation coefficient and *P* value. For the aquaplanet simulations, excluding the MPAS model (model 13) leads to an increased correlation coefficient to 0.54 that is statistically significant (*P* = 0.06). (b) Precipitation change in response to quadrupling CO_2_ relative to the control precipitation. The line corresponds to a 2.2%/K precipitation increase, which is obtained from a linear regression of the precipitation change on temperature change.

Figure 12 shows the climate and hydrological sensitivities in the aquaplanet and land simulations. Climate sensitivity is calculated as half of the global-mean surface temperature change between the Control and 4xCO2 simulations. In response to increased CO_2_ the models warm with climate sensitivities of 1.5–4.8 K (model median 3.3 K) in the aquaplanet setup. The tropical continent impacts climate sensitivity in a nonrobust way across models. In most models (AM2.1, CAM3, CAM4, CAM5Nor, MetUM-CTL, MetUM-ENT, MIROC5, and CALTECH) land does not strongly impact climate sensitivity, but in MPAS land increases climate sensitivity by 0.8 K and decreases it by 0.5–0.7 K in CNRM-AM5, ECHAM6.1, ECHAM6.3, and LMDZ5A. TRACMIP climate sensitivities are about as large as climate sensitivities reported with Earth system CMIP5 models in realistic setup, with a similar model spread [*Flato et al*., 2013]. This is despite the lack of positive radiative feedbacks from snow and sea ice and the lack of large continental landmasses that tend to warm more than ocean under global warming in realistic CMIP5 models [*Sutton et al*., 2007; *Byrne and O’Gorman*, 2013] and suggests that the TRACMIP setup includes a strong positive feedback that is missing from realistic CMIP5 simulations, a hypothesis that deserves more attention in future studies. There is a weak indication that some of the model spread in climate sensitivity results from differences in the control temperatures and an increase of climate sensitivity for warmer reference climate (Figure 12a), consistent with previous work [e.g., *Jonko et al*., 2013; *Caballero and Huber*, 2013; *Meraner et al*., 2013]. However, a statistically significant correlation between the control temperature and climate sensitivity is only found for the land simulations, and in the aquaplanet simulations if the MPAS model is excluded. As the models warm, global precipitation increases by 2.2% per degree K surface warming (Figure 12b), independent of the presence of land and in close agreement with realistic CMIP5 model simulations [*Held and Soden*, 2006; *Fläschner et al*., 2016].

**Figure 13.**
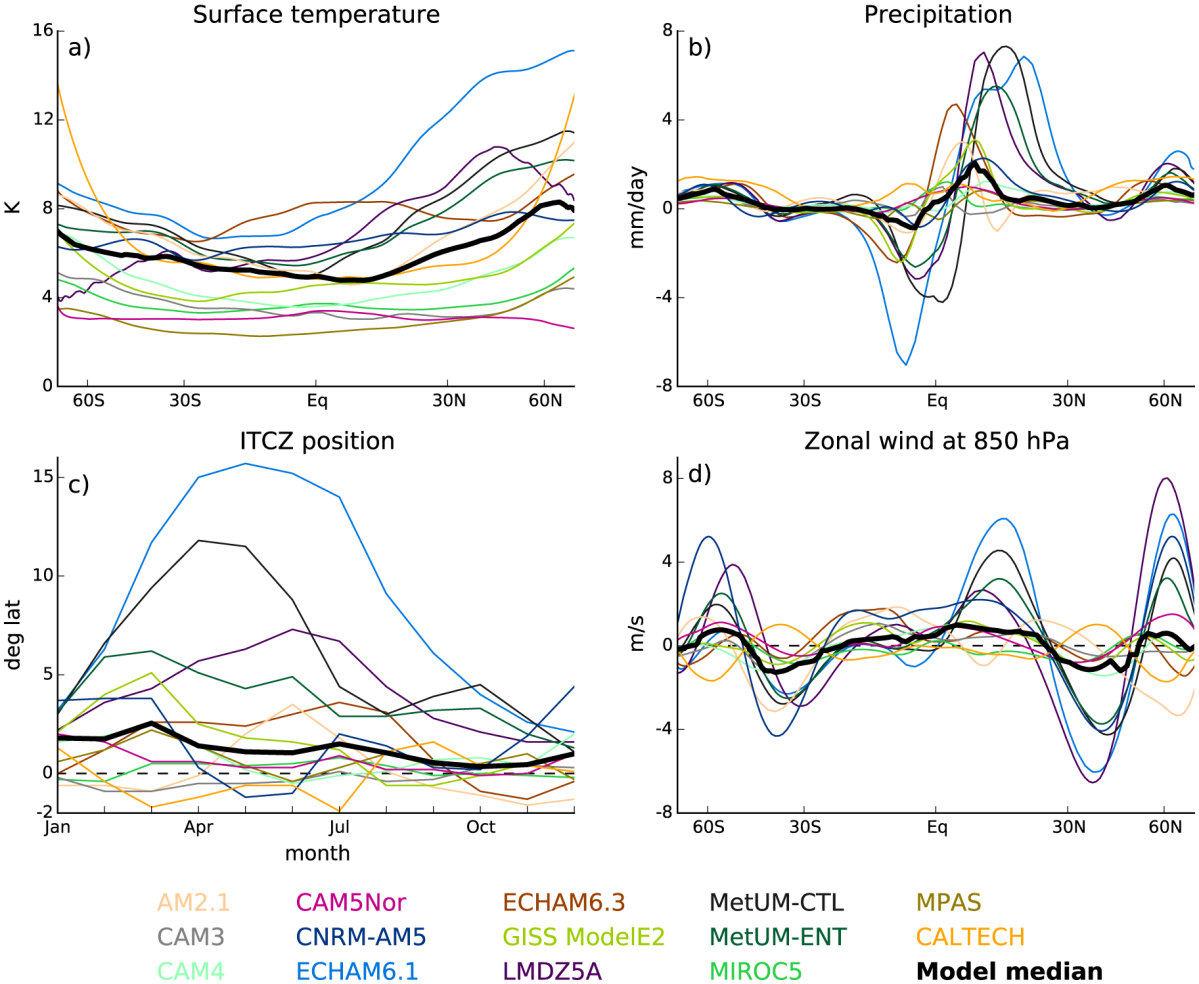
Response of the zonal-mean climate to a quadrupling of CO_2_ in the aquaplanet simulations. The difference between Aqua4xCO2 and AquaControl is shown. (a) Annual-mean surface temperature, (b) annual-mean precipitation, (c) seasonal evolution of the ITCZ position, and (d) annual-mean zonal wind at 850 hPa. Individual models are shown by the colored lines, the model median is shown by the thick black line.

More interesting is the spatial pattern of change, as revealed for the model median in Figures 5–7 and for individual models in Figure 13, which surveys the zonal mean climate change due to increasing CO_2_ in the aquaplanet simulations (Aqua4xCO2-AquaControl). In the model median, surface temperatures increase most strongly in high latitudes (Figure 13a), consistent with previous work [*Pithan and Mauritsen*, 2014] that has shown how high-latitude climate change is amplified due to local temperature feedbacks, even in the absence of albedo feedbacks (remember that there is no ice in any of the TRACMIP simulations). Yet polar amplification is much stronger in the Northern Hemisphere than in the Southern Hemisphere, and a handful of models do not produce any polar amplification in the Southern Hemisphere (e.g., the LMDZ5A model). The lack of strong warming around Antarctica in recent observed climate change and model projections for this century has been explained as primarily a transient response due to the slow heat uptake of a deep Southern Ocean [*Marshall et al*., 2014; *Armour et al*., 2016]. This is not what is happening in the TRACMIP simulations, as the anomalies are calculated at equilibrium and the prescribed slab ocean is a shallow 30 m globally. Instead, the explanation must lie with the balance of the atmospheric feedbacks that affect polar amplification: the water vapor, cloud, lapse-rate, and Planck feedbacks, as well as possible changes in atmospheric heat transport. This hypothesis is supported by the fact that polar amplification is equally strong in the Northern and Southern Hemisphere in the idealized CALTECH model that does not take into account radiative feedbacks from clouds and water vapor.

Under CO_2_ quadrupling, the median annual-mean precipitation in the deep tropics intensifies and becomes more sharply peaked around the Northern Hemisphere maximum (Figure 6). This pattern does not simply follow the wet-get-wetter paradigm [*Held and Soden*, 2006] but involves changes in tropical circulation (Figure 13b). In particular, the ITCZ shifts northward in essentially all models and all seasons (Figure 13c). The attendant mean meridional circulation is weakened outside the deep tropics (as expected from warming [*Held and Soden*, 2006]), but is strengthened in the northern deep tropics (Figure 7c), indicating a local strengthening of the ascending motion, consistent with the northern shift of the rain belt. The vertical reach of the Hadley cell is also increased, as expected, and the core of the subtropical jets is lifted in accordance. Changes in the tropospheric time mean zonal wind are relatively small in the model median, but the extratropical eddy-driven jet experiences substantial shifts in some models, leading to a large model spread (Figure 13d). The Northern hemisphere jet shifts poleward by −0.9 to +10.8° (model median +2.1°) and the Southern Hemisphere jet shifts poleward by +0.7 to +8.3° (model median +1.7°) in the comprehensive models. Interestingly, the idealized CALTECH model deviates from this picture, as it simulates a contraction instead of widening of the circulation (the Northern and Southern hemisphere jets shift equatorward by 5.4 and 5.0° lat, respectively). The contraction of the circulation in CALTECH is likely related to a much stronger warming of the poles in that model [*Butler et al*., 2010, Figure 13], and highlights the role that radiative interactions of clouds and water vapor play for the response of regional temperatures and the extratropical circulation to global climate change [*Voigt and Shaw*, 2015, 2016; *Ceppi and Hartmann*, 2016].

**Figure 14.**
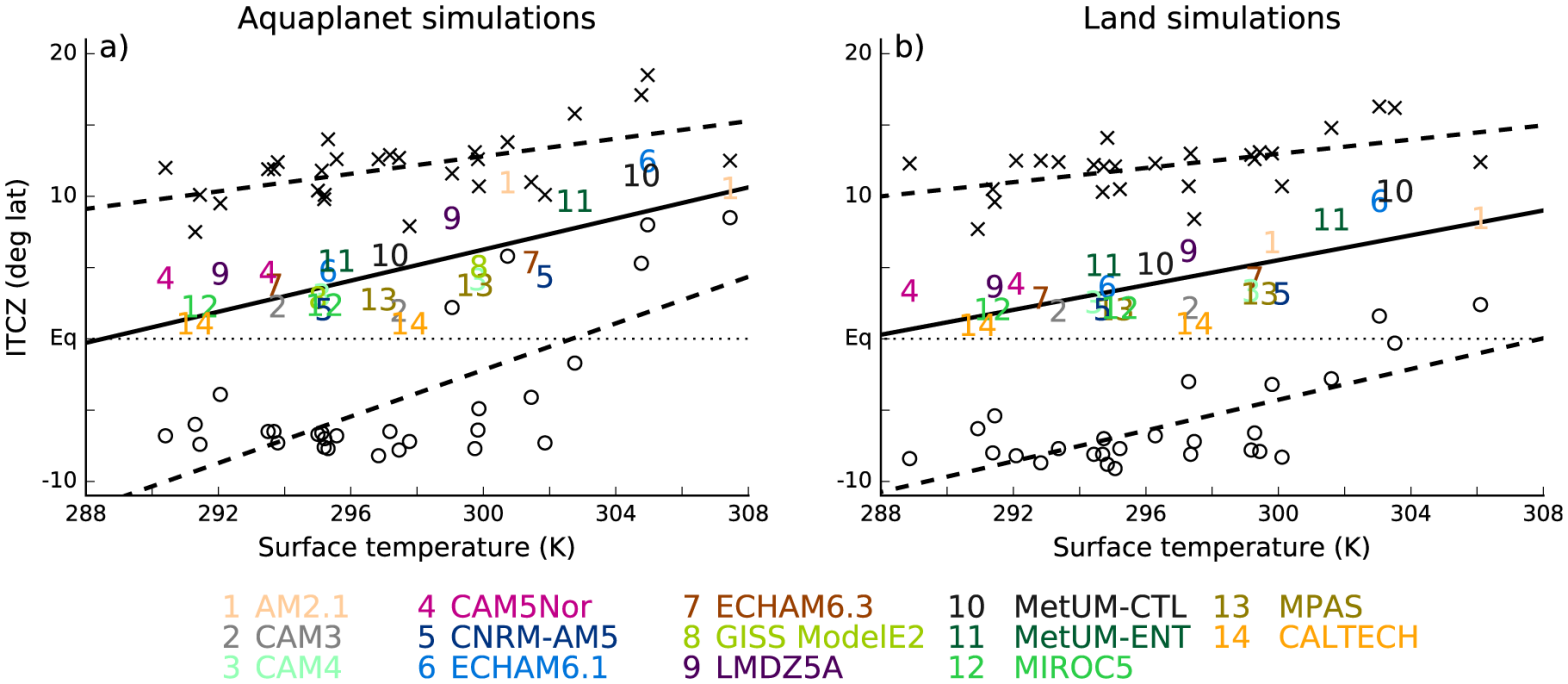
ITCZ position as a function of global-mean annual-mean surface temperature in (a) the aquaplanet simulations and (b) the simulations with land. The plot shows both the Control and 4xCO2 simulations. The numbers give the annual-mean ITCZ position for individual models, and the thick solid line is the linear regression of the annual-mean ITCZ position on surface temperature (regression slopes 0.55°/K for aquaplanet and 0.43°/K with land). For each model the 4xCO2 simulation is located to the right of the Control simulation. Moreover, the most northern ITCZ position occurring over the seasonal cycle is shown for each model and for both the Control and 4xCO2 simulation by the crosses, and the regression line through these most northern ITCZ excursions is shown by the upper dashed line (regression slopes 0.30°/K for aquaplanet and 0.24°/K with land). Similarly, the circles are the most southern ITCZ positions and the regression through these is shown by the lower dashed line (regression slopes 0.81°/K for aquaplanet and 0.54°/K with land).

Although the model spread in the ITCZ shift is considerable (Figure 13c and Table 4), it is clear that the annual-mean northward shift of the ITCZ is mostly accomplished by anomalies during the first half of the year, when the ITCZ is in the Southern Hemisphere in the control climate. That is, excursions of the ITCZ into the Southern Hemisphere are weakened in a warmer world. To verify if this connection between warmer temperatures and a more northerly confinement of the ITCZ holds across models and basic state, we have plotted the annual-mean and seasonal excursion of the ITCZ as a function of global-mean temperature in AquaControl and Aqua4xCO2 simulations (Figure 14a) and in LandControl and Land4xCO2 simulations (Figure 14b) (we obtain the same qualitative result when we plot the ITCZ versus annual-mean tropical-mean surface temperature). Indeed, in both setups, the annual-mean position of the ITCZ (colored numbers indicating single experiments) is further north the warmer the global-mean temperature and this shift is mostly accomplished through a northward shift of the southernmost seasonal reach of the ITCZ (open dots). The northernmost reach of the ITCZ also shifts north with a warmer climate, although the response is more modest. The same qualitative behavior is seen with or without the presence of the tropical continent, but the migration of the southern edge is less steep with land (0.54°/K, as opposed to 0.81°/K for Aqua simulations). Contractions in the width of the ITCZ have also been observed in other circumstances, models, and setups. These studies have suggested several mechanisms by which convective zones might shrink with a warming climate, including increased upper tropospheric static stability [*Bony et al*., 2016], an upped-ante for convection because of low-level inflow of relatively dry air [*Neelin et al*., 2003], cloud-radiative changes [*Voigt and Shaw*, 2015], and changes in energy transport by the Hadley circulation and transient eddies [*Byrne and Schneider*, 2016]. It remains to be investigated whether one or several of these mechanisms explain the contraction seen in TRACMIP. The reason why the southern edge migrates more than the northern edge might be a different balance of these mechanisms, it might reflect a dynamic limit to how far poleward the northward edge of the ITCZ can migrate in TRACMIP given Earth’s rotation rate (S. Faulk et al., Dynamical constraints on the ITCZ extent in planetary atmospheres, submitted to *Journal of the Atmospheric Sciences*, 2016), or it might be better understood in terms of warmer-get-wetter and seasonal amplification mechanisms [*Huang et al*., 2013].

**Figure 15.**
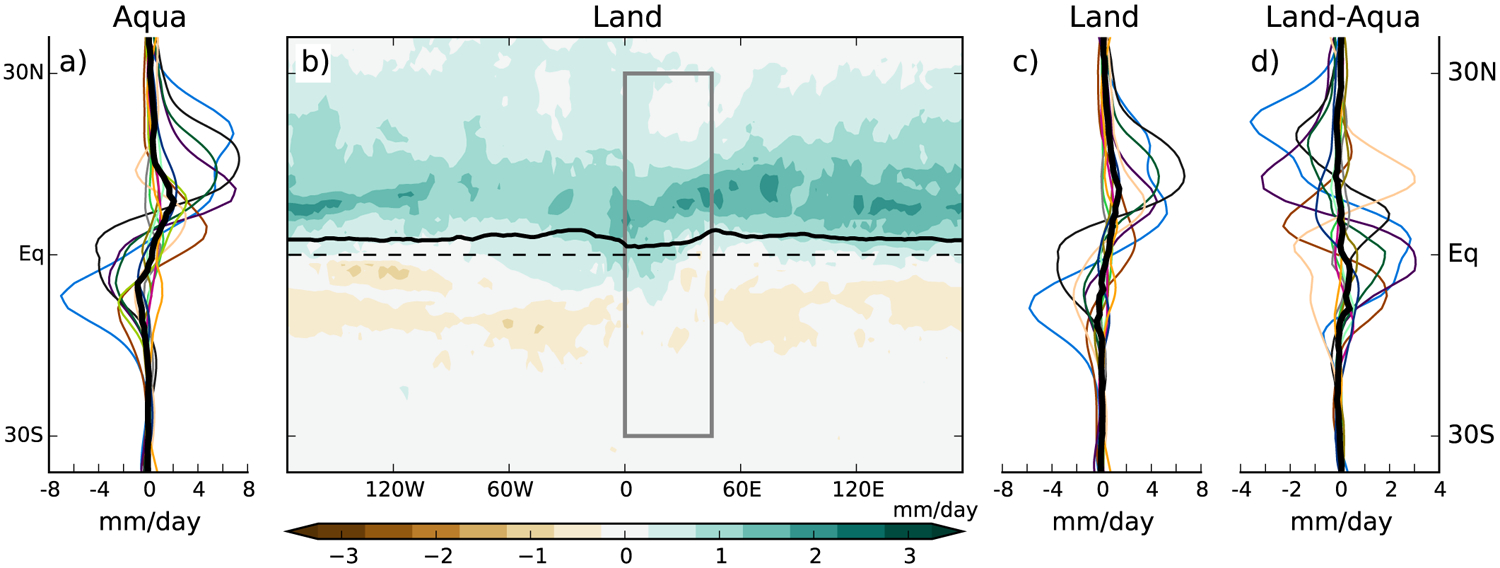
Annual-mean precipitation response between 40°N and 40°S to increased CO_2_ in aquaplanet and land simulations. (a) Zonal-mean response in the aquaplanet setup, (b) longitude-latitude response of the model-median precipitation in the land setup, (c) zonal-mean response in the land setup, and (d) difference between zonal-mean response in the land versus aquaplanet setup. In Figures 15a, 15c, and 15d models are colored according to the color coding introduced in Figure 2; the model median is shown by the thick black line. In Figure 15b, the black line is the model-median ITCZ in LandControl.

One motivation for TRACMIP is to investigate if the breaking of the zonal symmetry by land changes not only the basic state of tropical precipitation, but also its sensitivity to external forcings. We have mentioned above how the ITCZ in the Land simulations displays the same qualitative behavior as in the Aqua simulations, but that the displacement of the southern edge was weaker. In Figure 15 we show in a more quantitative way how the annual-mean zonal-mean precipitation response to CO_2_ quadrupling is affected by the presence of land.

Figure 15b shows how CO_2_ forces a northward shift of the model-median rainband at all longitudes, including over the “jello” continent. The model-median anomalies tend to be slightly stronger to the east of the continent, but there is no model-robust signal in the zonal distribution of the anomalies. For example, the precipitation increases most strongly over the Northern Hemisphere continent in CAM4 and CAM5Nor, but over the Northern Hemisphere ocean in ECHAM6.1 and ECHAM6.3 (not shown). These differences likely arise from differences in the LandControl climate as well as a different response of clouds and convection. Irrespective of the lack of a robust signal in the zonal pattern, models agree qualitatively on how land impacts the zonal-mean precipitation response. In the zonal-mean, the anomalous rainfall dipole under increased CO_2_ is similar in the Aqua and Land simulation, both in terms of median location and model spread (compare Figures 15a and 15c). However, the magnitude of the response in the Land simulations is substantially muted and the ITCZ shift is smaller in many of the models (Table 4), even though land only occupies 6% of Earth’s surface in TRACMIP (12% of the tropical longitudes). This reduction is a consequence of both the weak anomalies over the continent itself and weaker anomalies over the ocean, especially downstream from the continent (see also Figures 6c and 6d). The presence of land thus strongly modulates the sensitivity of the ITCZ to changes in CO_2_ even when only a small percentage of Earth is covered by land (5 times less than in the present-day climate). While more work is needed to understand this behavior, it suggests that caution should be applied when using idealized aquaplanet simulations to understand the sensitivity of present-day climate to greenhouse gas forcing.

### 4.2. Response to Changes in Insolation

We now briefly describe the response to a change in seasonal insolation by comparing the LandControl and LandOrbit simulations. The seasonal insolation change was shown in Figure 3. Insolation is reduced during boreal summer (JJA) and increased during austral summer (DJF), leading to less seasonal insolation contrast in the Northern Hemisphere in LandOrbit compared to LandControl and more seasonal insolation contrast in the Southern Hemisphere. The insolation change of LandOrbit-LandControl is similar to the insolation change between present-day and the mid-Holocence.

**Figure 16.**
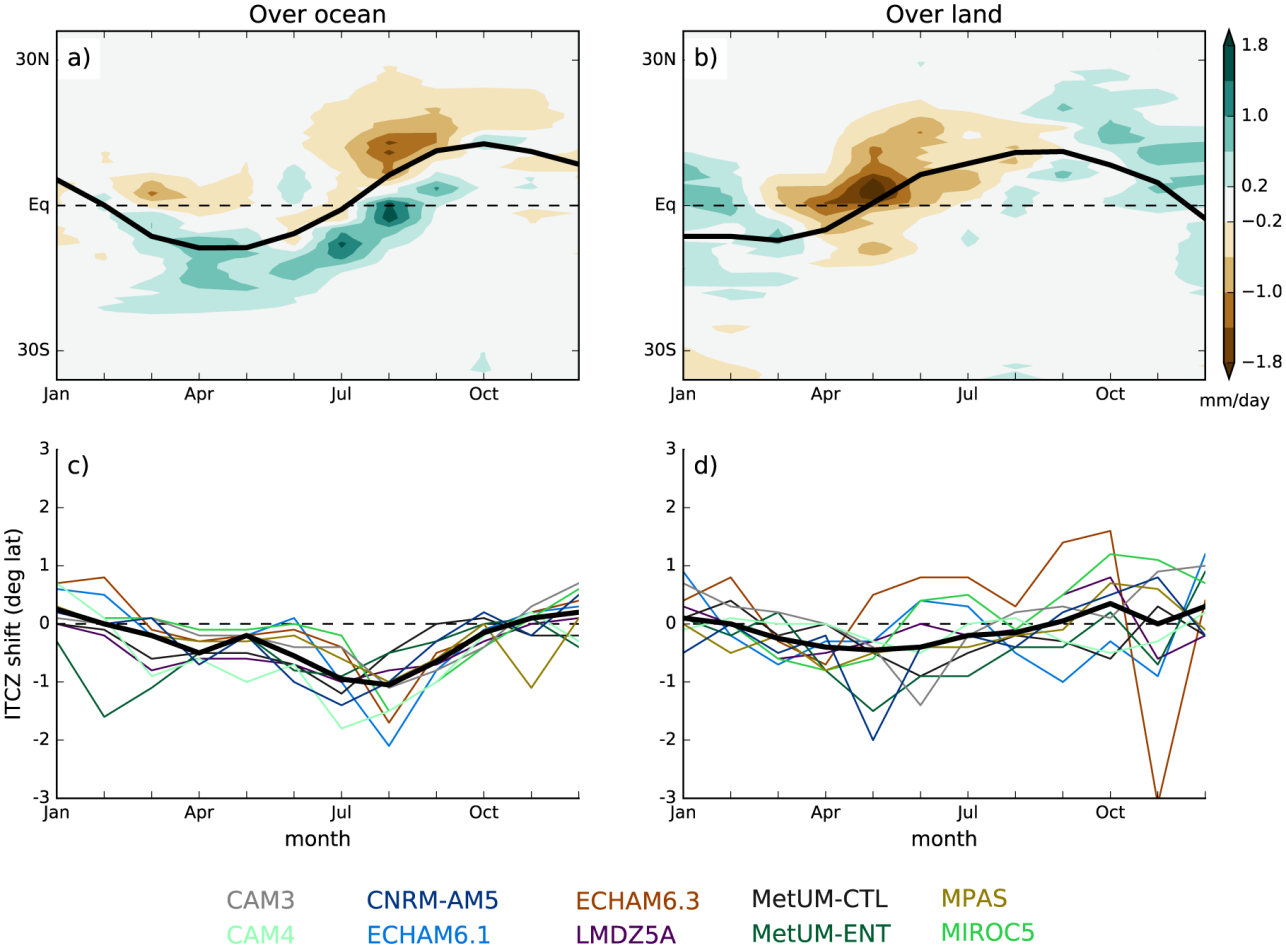
Response of seasonal precipitation and ITCZ location to a seasonal insolation change (LandOrbit-LandControl) averaged zonally over ocean longitudes (180°W–0°E, 45°E–180°E; left) and land longitudes (0°E–45°E; right). For Figures 16a and 16b the model median is shown and the black lines are the model median LandControl ITCZ calculated over ocean and land longitudes, respectively. For Figures 16c and 16d, the thick black line is the model median change.

Figure 16 shows the change of seasonal precipitation and ITCZ location separately calculated over ocean and land. In the model median, the orbital forcing causes ocean precipitation to increase south of the control ITCZ (black line in Figure 16a) and to decrease to the north of it from March through September (marginally in October). As a result, the ocean ITCZ shifts southward during these months in almost all models (Figure 16c). The rest of the year, the oceanic precipitation and the ITCZ position show little change in the model median, and the ITCZ shift is not robust across models. The response of land precipitation and ITCZ is qualitatively different from the response over ocean (Figures 16b and 16d). Model-median precipitation changes are concentrated to the north of the climatological rainband and tend to be symmetric with respect to the equator, with reduced precipitation during March–July (roughly when insolation is reduced) and increased precipitation during October–February (roughly when insolation is increased). This is consistent with insolation changes driving changes in monsoonal circulations. In contrast to ocean regions, and consistent with the model differences in the Land simulation, the land ITCZ does not shift robustly across models in any of the seasons, and models differ in the direction of the land ITCZ shift.

In the annual-mean zonal-mean (average over all longitudes), all models shift their ITCZ southward (−0.2 to −1.0° lat; model median −0.6° lat; Table 4). Importantly, the zonal mean shift is dominated by a robust southward shift over the ocean (−0.4 to −0.7° lat; model median −0.6° lat), while the ITCZ response over land is not robust across models (0.2 to −0.7° lat; model median −0.2° lat). This suggests that zonal-mean frameworks of atmospheric energetics and ITCZ shifts are not sufficient to understand past regional precipitation changes, such as the greening of the Sahara during the early and mid-Holocene (11,000–5000 BPE) [*Hoelzmann et al*., 1998; *Kuper and Kröpelin*, 2006]. We hope that the TRACMIP orbital simulations, in combination with the quadrupled CO_2_ simulations, will prove helpful to understand how zonal mean frameworks can be extended to understand past and future regional changes.

## 5. Conclusions

This paper has presented the new Tropical Rain belts with an Annual cycle and Continent-Model Intercomparison Project, TRACMIP. TRACMIP is a community effort that is motivated by the desire to better understand the dynamics of tropical rain belts, how they respond to internal (seasonal and diurnal insolation cycles) and external (CO_2_ and orbital changes) forcings, and how zonal-mean frameworks can be extended to understand regional rain belt changes. The suite of TRACMIP experiments includes an aquaplanet configuration and one with an idealized tropical continent. In all cases the lower boundary allows for thermodynamic coupling with the atmosphere and a closed surface energy balance, an important factor for modeling tropical rainfall [*Kang and Held*, 2012]. TRACMIP thus fills the gap in the CMIP5 model hierarchy between the fixed-SST aquaplanet simulation and coupled simulations in realistic setups. Accordingly, the TRACMIP simulations are much closer than the CMIP5 fixed-SST aquaplanets to the observed tropical rainfall and global circulation patterns, suggesting that they can be used as a simple analog to realistic model configurations.

In this survey of the main aspects of the TRACMIP simulations we have focused mostly on the ensemble mean response of the monthly climatology or of the annual mean to changes in configuration and external forcings. We have highlighted how the presence of a “jello” continent changes both the basic state and the sensitivity to greenhouse forcing of oceanic areas away from the continent, how quadrupling CO_2_ leads to an amplification of the asymmetry between the Northern and the Southern Hemispheres both in temperature and precipitation, and how differently land and ocean respond to changes in the seasonality of insolation. The spread across models is, nonetheless, just as interesting. In particular, we have noted how the range of climate sensitivity is as large for the aquaplanets as it is for standard CMIP5 simulations and how the scatter would seem to suggest that a warmer climate is also a more sensitive climate. The way in which the simplified climate model differs from the comprehensive models is a testament to the importance of clouds and convective processes. These and many other aspects of the ensemble scatter invite deeper investigations. For example, why is the land response to orbital changes much less robust than the oceanic changes? What makes one climate model an outlier by one measure, but not by any other?

In this paper, we have only presented results that relate to seasonal or longer timescales, but TRACMIP data can be used to investigate aspects of the climate that range from the diurnal cycle to the synoptic scale and the scale of intraseasonal variability. And although the original motivation for TRACMIP was the investigation of the tropical rain belts, its use extends beyond this topic. Indeed, we hope that TRACMIP will also resonate with the extratropical community and will provide a helpful perspective on extratropical jet streams and storm tracks. TRACMIP is an ongoing effort that combines a hierarchy of boundary conditions with a hierarchy of climate models. As such we hope that the community that has coalesced around this project will grow in both contributors and users.

## Figures and Tables

**Table 1.** Five Experiments Explore the Dynamics of Tropical Rainbands (Monsoons and ITCZ)^a^

Experiment	Land	CO_2_ (ppmv)	Eccentricity *ϵ*	Years	Initial Condition
AquaControl	No	348	0	15 + 30	Arbitrary initial state, 15 years of spin-up
LandControl	Yes	348	0	40	Year 45 of AquaControl
Aqua4xCO2	No	1392	0	40	Year 45 of AquaControl
Land4xCO2	Yes	1392	0	40	Year 40 of LandControl
LandOrbit	Yes	348	0.02	40	Year 40 of LandControl

a The control configuration is an aquaplanet coupled to a slab ocean. Insolation varies with diurnal and annual cycles. CO2 is varied, and a tropical jello-continent and different orbital parameters are used to study tropical rainfall in present-day-like conditions and in conditions mimicking the mid-Holocene and global warming.

**Table 2.** Coefficients for the Fourth-Order Polynomial Fit of the TRACMIP q-Flux to the Observed q-Flux (equation (3))^a^

	Northern Hemisphere	Southern Hemisphere
*p*_0_	−50.1685 (aquaplanet)	−56.0193 (aquaplanet)
	−50.7586 (land)	−56.6094 (land)
*p*_1_	4.9755	−6.4824
*p*_2_	−1.4162 × 10^−1^	−2.3494 × 10^−1^
*p*_3_	1.6743 × 10^−3^	−3.4685 × 10^−3^
*p*_4_	−6.8650 × 10^−6^	−1.7732 × 10^−5^

a Simulations with land include a small correction of the q-flux over ocean, which is implemented by a spatially uniform decrease of *p*_0_ compared to the aquaplanet simulation.

**Table 3.** Climate Models Participating in TRACMIP

	Model	Reference	Resolution	Remarks
1	AM2.1	*Anderson et al*. [2004], *Lin* [2004], and *Delworth et al.* [2006]	2.0° lat × 2.5° lon, finite-volume dynamical core; 24 levels	Atmosphere component of GFDL-CM2.1
2	CAM3	*Collins et al*. [2006]	T42 (2.8°); 26 levels	Atmosphere component of CCSM3
3	CAM4	*Neale et al.* [2013]	1.9° lat × 2.5° lon, finite volume (nominally 2.0°); 26 levels	Atmosphere component of CCSM4, aquaplanet modifications as in *Rose et al*. [2014]
4	CAM5Nor	*Kirkevåg et al.* [2013]	2.5 × 1.9°; 30 levels	Based on CAM5.3/CESM1.2; aerosol physics and chemistry components of CAM-Oslo [*Kirkevag et al.*,2013]; energy updates of *Williamson et al.* [2015]; computation for air-sea fluxes as in *Fairall et al.* [2013]; modified dynamical core for improved conservation of angular momentum and global fixer (T. Toniazzo, personal communication, 2016)
5	CNRM-AM5	*Voldoire et al.* [2013]	T127 (0.9° lat × 0.9° lon); 31 levels	Atmosphere component of CNRM-CM5
6	ECHAM6.1	*Stevens et al*. [2013]	T63 (1.9° lat × 1.9° lon), 47 levels	Atmosphere component of MPI-ESM
7	ECHAM6.3	*Stevens et al*. [2013]	T63 (1.9° lat × 1.9° lon), 47 levels	Update of ECHAM6.1
8	GISS ModelE2	*Schmidt et al*. [2014b] and *Miller et al*. [2014]	2.0° lat × 2.5° lon; 40 levels	Atmosphere-only version of GISS ModelE2
9	LMDZ5A	*Hourdin et al*. [2013]	1.9° lat × 3.8° lon; 39 levels	Atmosphere component of IPSL-CM5A-LR
10	MetUM-CTL	*Walters et al.* [2016]	N96 (1.9° lat × 1.3° lon); 85 levels	Standard configuration of GA6.0
11	MetUM-ENT	*Walters et al.* [2016]	N96 (1.9° lat × 1.3° lon); 85 levels	+50% convective entrainment and detrainment compared to MetUM-CTL
12	MIROC5	*Watanabe et al.* [2010]	T85 (1.4° lat × 1.4° lon); 40 levels	
13	MPAS	*Skamarock et al*. [2012]	240 km; 30 levels	
14	CALTECH	*O’Gorman and Schneider* [2008] and *Bordoni and Schneider* [2008]	T42 (2.8°); 30 levels	Idealized physics: gray radiation, no radiative effects of clouds and water vapor, simplified Betts-Miller convection scheme

**Table 4.** Global-Mean Time-Mean Surface Temperature (in Units of K) and ITCZ Position in the Various TRACMIP Experiments^a^

	Model	AquaControl	LandControl	Aqua4xCO2	Land4xCO2	LandOrbit
1	AM2.1	300.7/10.8°	299.8/6.6°	6.7/−0.4°	6.3/1.7°	
2	CAM3	293.8/2.1 °	293.4/1.8°	3.7/−0.3°	4.0/0.2°	−0.2/−0.4°
3	CAM4	295.1/2.9°	294.4/2.4°	4.7/1.1 °	4.7/0.8°	0.1/−0.2°
4	CAM5Nor	290.4/4.1 °	288.9/3.2°	3.1/0.4°	3.2/0.5°	
5	CNRM-AM5	295.2/1.9°	294.7/1.9°	6.7/2.3°	5.4/1.1 °	0.1/−0.5°
6	ECHAM6.1	295.3/4.6°	294.8/3.5°	9.6/7.7°	8.2/6.0°	0.1/−0.6°
7	ECHAM6.3	293.7/3.6°	292.8/2.7°	7.8/1.6°	6.5/1.4°	0.0/−0.6°
8	GISS ModelE2	295.0/2.7°		4.9/2.2°		
9	LMDZ5A	292.1/4.4°	291.4/3.5°	7.0/3.9°	5.9/2.5°	0.0/−0.6°
10	MetUM-CTL	297.2/5.7°	296.3/5.1 °	7.6/5.6°	7.2/5.1 °	0.1/−0.7°
11	MetUM-ENT	295.6/5.3°	294.7 5.0°	7.2/4.2°	6.9/3.2°	0.2/−1.0°
12	MIROC5	291.4/2.1 °	291.4/1.9°	3.8/0.1 °	3.8/0.1 °	0.0/−0.3°
13	MPAS	296.8/2.6°	295.1/1.9°	2.9/1.0°	4.4/1.1 °	0.0/−0.2°
14	CALTECH	291.3/0.9°	290.9/0.8°	6.5/0.0°	6.5/0.1 °	
	Model median	295.1/3.3°	294.4/2.7°	6.6/1.4°	5.9/1.1 °	0.0/−0.6°

a The ITCZ position is calculated from the zonal-mean time-mean precipitation as the latitude of the precipitation centroid between 30°N and 30°S. The Aqua4xCO2 values are given as the change with respect to AquaControl, and the Land4xCO2 and LandOrbit values as the change with respect to LandControl.

## Key Points:

- TRACMIP is a new model intercomparison project on tropical rain belt dynamics in today’s, past, and future climate
- Idealized simulations with comprehensive models coupled to slab ocean fill gap in current model hierarchy
- This paper surveys TRACMIP’s broad climate characteristics and highlights promising research directions
